# The Generalized Stochastic Smoluchowski Equation

**DOI:** 10.3390/e21101006

**Published:** 2019-10-15

**Authors:** Pierre-Henri Chavanis

**Affiliations:** Laboratoire de Physique Théorique, Université de Toulouse, CNRS, UPS, 31000 Toulouse, France; chavanis@irsamc.ups-tlse.fr

**Keywords:** Fokker-Planck equations, free energy, generalized thermodynamics, instantons, dynamical density functional theory

## Abstract

We study the dynamics of a system of overdamped Brownian particles governed by the generalized stochastic Smoluchowski equation associated with a generalized form of entropy and involving a long-range potential of interaction [P.H. Chavanis, Entropy **17**, 3205 (2015)]. We first neglect fluctuations and provide a macroscopic description of the system based on the deterministic mean field Smoluchowski equation. We then take fluctuations into account and provide a mesoscopic description of the system based on the stochastic mean field Smoluchowski equation. We establish the main properties of this equation and derive the Kramers escape rate formula, giving the lifetime of a metastable state, from the theory of instantons. We relate the properties of the generalized stochastic Smoluchowski equation to a principle of maximum dissipation of free energy. We also discuss the connection with the dynamical density functional theory of simple liquids.

## 1. Introduction

The theory of Brownian motion started with the pioneering work of Einstein [1,2] (as noted in [3], the premices of the theory of Brownian motion may be traced back to the beautiful paper of Lord Rayleigh [4] on the dynamics of massive particles bombared by numerous small projectiles). He showed that the probabilistic motion of a free Brownian particle in the overdamped limit can be described by the ordinary diffusion equation

(1) $$\begin{array}{c} \frac{\partial \rho }{\partial t}=D\Delta \rho , \end{array}$$

where ρ(r,t) denotes the probability density of finding the particle in r at time *t*. He also established the following relation

(2) $$\begin{array}{c} D=\frac{\chi k_{B}T}{m} \end{array}$$

between the diffusion coefficient, the mobility χ/m=1/ξm of the Brownian particle (*m* is the mass of the particle and ξ is the friction coefficient) and the temperature *T*. The same relation was obtained independently by Sutherland [5]. It was also re-derived by Langevin [6] in a different manner. The Einstein relation from Equation (2) is a simple example of the fluctuation–dissipation theorem in statistical mechanics. Smoluchowski [7] considered an overdamped Brownian particle in a fixed external potential $\Phi_{\mathrm{ext}}\left(r\right)$ and showed that the probability density ρ(r,t) satisfies the drift–diffusion equation

(3) $$\begin{array}{c} \frac{\partial \rho }{\partial t}=\nabla \cdot \left(D\nabla \rho +\chi \rho \nabla \Phi_{\mathrm{ext}}\right). \end{array}$$

The Smoluchowski Equation (3) is a particular example of Fokker-Planck equations [8,9,10,11]. It describes the diffusive motion of a Brownian particle (or an ensemble of non-interacting Brownian particles) in an external potential. Since the particles are coupled to a thermal bath fixing the temperature, the relevant statistical ensemble is the canonical ensemble. If we identify the equilibrium state of the Smoluchowski equation with the Boltzmann distribution

(4) $$\begin{array}{c} \rho_{\mathrm{eq}}\left(r\right)=A\;e^{-\beta m\Phi_{\mathrm{ext}}\left(r\right)}, \end{array}$$

where $\beta =1/k_{B}T$ is the inverse temperature, we immediately obtain the Einstein relation, Equation (2). The Smoluchowski equation satisfies an *H*-theorem for the Boltzmann free energy and relaxes towards the Boltzmann distribution from Equation (4). The Boltzmann distribution minimizes the Boltzmann free energy under the normalization condition.

We now consider an ensemble of Brownian particles interacting via a binary potential $u(|r-r^{\prime }|)$. For simplicity, we consider a potential with long-range interactions. For such potentials the mean field approximation becomes exact in a proper thermodynamic limit N→+∞ (see reviews in [12,13]). This is the situation considered in [14]. Starting from the *N*-body Smoluchowski equation, writing the first equation of the canonical BBGKY hierarchy and implementing the mean field approximation, which amounts to neglecting correlations, we find that the evolution of the average density of particles ρ(r,t) is governed by the nonlinear mean field Smoluchowski equation [14]:

(5) $$\begin{array}{c} \frac{\partial \rho }{\partial t}=\nabla \cdot \left(D\nabla \rho +\chi \rho \nabla \Phi \right), \end{array}$$

where the potential Φ(r,t) is given by

(6) $$\begin{array}{c} \Phi \left(r,t\right)=\int \rho \left(r^{\prime },t\right)u(|r-r^{\prime }\left|\right)\;dr^{\prime }. \end{array}$$

Contrary to systems described by the ordinary Smoluchowski Equation (3) where the Brownian particles move in a fixed external potential $\Phi_{\mathrm{ext}}\left(r\right)$, in the present situation the Brownian particles move in a mean field potential Φ(r,t) that they create themselves. The equilibrium states of the mean field Smoluchowski equation are given by the mean field Boltzmann distribution [14]:

(7) $$\begin{array}{c} \rho_{\mathrm{eq}}\left(r\right)=A\;e^{-\beta m\Phi_{\mathrm{eq}}\left(r\right)}, \end{array}$$

where $\Phi_{\mathrm{eq}}\left(r\right)$ is related to $\rho_{\mathrm{eq}}\left(r\right)$ through Equation (6). This leads to the self-consistency relation

(8) $$\begin{array}{c} \rho_{\mathrm{eq}}\left(r\right)=A\;e^{-\beta m\int \rho_{\mathrm{eq}}\left(r^{\prime }\right)u(|r-r^{\prime }\left|\right)\;dr^{\prime }}. \end{array}$$

This integral equation may have several solutions. Therefore, different equilibrium states (stable, unstable and metastable) are possible. The mean field Smoluchowski Equation (5) satisfies an *H*-theorem for the mean field Boltzmann free energy and relaxes towards a stable equilibrium state, given by the mean field Boltzmann distribution from Equation (7), which is a (local) minimum of the Boltzmann free energy at fixed mass. When several stable solutions exist, their selection depends on a notion of basin of attraction [14]. The mean field Smoluchowski Equation (5) appeared in different domains of physics such as electrolytes [15,16,17,18], systems with long-range interactions [14,19,20,21,22,23], bacterial populations undergoing chemotaxis [24], kinetic theory of adsorbates [25], superconductors [26,27], self-gravitating Brownian particles [28,29,30,31,32,33,34,35,36,37,38,39,40,41,42,43], directed self-assembly of nanoparticles [44], 2D Brownian vortices [45,46], colloids at a fluid interface [47], nucleation [48], the Brownian mean field (BMF) model [49], etc. These models are reviewed in [50]. The Smoluchowski Equation (5) also describes the dynamics of simple liquids, for which correlations must be taken into account, provided that the potential of interaction $u(|r-r^{\prime }|)$ is replaced by an effective potential of interaction $u_{\mathrm{eff}}(|r-r^{\prime }\left|\right)=-\frac{k_{B}T}{m^{2}}c(|r-r^{\prime }\left|\right)$ related to the direct correlation function $c(|r-r^{\prime }|)$ [51,52,53,54,55,56,57,58,59,60,61,62,63] (for ultrasoft particles the mean field approximation applies and $u_{\mathrm{eff}}(|r-r^{\prime }\left|\right)=u(|r-r^{\prime }\left|\right)$ [64]). In this context, the self-consistency relation from Equation (8) was first established by Kirkwood and Monroe [65,66]. The Smoluchowski equation is valid in the strong friction limit ξ→+∞. More general equations taking inertial effects into account are considered in [14,23,63,67,68].

The mean field Smoluchowski Equation (5) is a deterministic equation that ignores fluctuations. If we now take fluctuations into account and use the theory of fluctuating hydrodynamics developed by Landau and Lifshitz [69], we obtain the stochastic mean field Smoluchowski equation [14]:

(9) $$\begin{array}{c} \frac{\partial \rho }{\partial t}=\nabla \cdot \left(D\nabla \rho +\chi \rho \nabla \Phi \right)+\nabla \cdot \left(\sqrt{2\chi k_{B}T\rho }\;R\left(r,t\right)\right), \end{array}$$

(10) $$\begin{array}{c} \Phi \left(r,t\right)=\int \rho \left(r^{\prime },t\right)u(|r-r^{\prime }\left|\right)dr^{\prime }, \end{array}$$

where R(r,t) is a Gaussian white noise satisfying 〈R(r,t)〉=0 and $\langle R_{i}\left(r,t\right)R_{j}\left(r^{\prime },t^{\prime }\right)\rangle =\delta_{ij}\delta \left(r-r^{\prime }\right)\delta \left(t-t^{\prime }\right)$. The stochastic mean field Smoluchowski Equation (9), which is a partial differential stochastic equation, may be interpreted as a Langevin equation for the density field ρ(r,t). As we have previously indicated, the deterministic mean field Smoluchowski Equation (5) may have several stable equilibrium states. In the absence of fluctuations, the system relaxes towards one of these states and stays there permanently. In the presence of fluctuations, the system undergoes random transitions from one stable state to the other. Such switches can be described in terms of the stochastic mean field Smoluchowski Equation (9) including a random forcing due to fluctuations [14]. The stochastic term allows the system to jump from one equilibrium state to another. These properties have been illustrated numerically by Chavanis and Delfini [70] for a model of self-gravitating Brownian particles and bacterial populations presenting two symmetric metastable states. In that case, the system experiences random transitions from one state to the other. The lifetime of the metastable states is related to the Kramers escape rate formula which can be derived from the theory of instantons [70]. To the best of our knowledge, the stochastic Smoluchowski Equation (9) was first written down in van Kampen’s book [71] (p. 349) referring to the papers of van Vliet [72,73] in 1971; this is the oldest reference to that equation that we have found. It then appeared in different domains of physics such as the theory of simple liquids [63,74,75,76,77,78], the kinetic theory of adsorbates [25], systems with long-range interactions [14], 2D Brownian vortices [45], bacterial populations undergoing chemotaxis [79], nucleation [48], self-gravitating Brownian particles [70], the BMF model [49], etc. It can also be exported to other systems (electrolytes, superconductors, directed self-assembly of nanoparticles, colloids at a fluid interface, etc.) as discussed in [50]. More general stochastic partial differential equations taking inertial effects into account are considered in [14,63,79].

An important topic in statistical mechanics and kinetic theory that emerged over the last decades concerns the notion of generalized thermodynamics pioneered by Tsallis [80]. It has been observed in many occasions that the Boltzmann entropy does not provide a correct description of the system under consideration and that other forms of entropies may be more relevant. In the context of Brownian motion, the Boltzmann distribution emerges naturally from the Smoluchowski equation when the particles have a constant diffusion coefficient and a constant mobility. This corresponds to an “ideal” situation where the particles do not experience microscopic constraints. However, one may encounter “complex” situations in which the particles are hampered in their motion by some small-scale constraints (due to short-range interactions) so that the diffusion coefficient and the mobility depend on the local density. In that case, the motion of the particles is biased, resulting in anomalous diffusion or anomalous mobility. The corresponding generalized Smoluchowski equation satisfies a canonical *H*-theorem for a generalized free energy and relaxes towards an equilibrium state, different from the Boltzmann distribution, which minimizes the generalized free energy at fixed mass. Initially, generalized thermodynamics and generalized Fokker-Planck equations were introduced in relation to the Fermi–Dirac [81,82], Bose–Einstein [82,83,84] and Tsallis [85,86,87,88,89,90] entropies. This gave the impression that these entropies were special and that a generalized thermodynamical formalism could be developed only for these functionals. However, it was shown later by [50,91,92,93,94,95,96,97,98,99,100,101,102,103,104,105,106,107] that generalized thermodynamics and nonlinear Fokker-Planck equations could be extended to arbitrary forms of entropic functionals (see reviews in [50,106,108,109]). In the situations described above, generalized entropies arise because the system experiences small-scale constraints so that some of the microstates are forbidden [50]. (As far as we know, the first “generalized entropy” was introduced in 1908 by Ornstein [110] in relation to the van der Waals’ equation of state (see van Kampen [111]). This entropy can be obtained from a combinatorial analysis taking into account excluded volume effects. On the other hand, as discussed in [112], a functional equivalent to the Tsallis entropy was introduced long ago in astrophysics by Ipser [113] in a dynamical (Vlasov) context in relation to Eddington’s stellar polytropes [114]. Ipser [113] also introduced functionals of the form S=−∫C(f)drdv (where C(f) is an arbitrary convex function of the distribution function f(r,v)) that we now call “generalized entropies” [106]. Similar functionals (for the coarse-grained distribution function $\bar{f}\left(r,v\right)$) were introduced by Antonov [115] who called them “quasi-entropies” (see reference [116]) and, independently, by Tremaine et al. [117] who called them “*H*-functions”.)

If we combine generalized thermodynamics [109], long-range interactions [12,13] and fluctuating hydrodynamics [69], we obtain a generalized stochastic mean field Smoluchowski equation [50] given by Equation (83) having a source of nonlinearity arising from the self-consistent mean field potential (long-range interactions), a source of nonlinearity arising from the fact that the diffusion coefficient and the mobility of the particles depend on the density itself (generalized thermodynamics) and a stochastic (noise) term taking fluctuations into account (fluctuating hydrodynamics). In this paper, we present a brief overview of this important equation stressing its main properties.

The paper is organized as follows. In Section 2, we consider a system of overdamped Brownian particles with long-range interactions and show how a notion of generalized thermodynamics emerges in the case where these particles experience small-scale constraints. In Section 3, we neglect fluctuations and consider the generalized deterministic mean field Smoluchowski equation describing the macroscopic dynamics of these systems. In Section 4, we take fluctuations into account and consider the generalized stochastic mean field Smoluchowski equation describing the mesoscopic dynamics of these systems. We derive the Kramers escape rate formula, giving the lifetime of a metastable state, from the theory of instantons and make the connection with the maximum free energy dissipation principle.

## 2. Generalized Thermodynamics

### 2.1. Generalized Free Energy

We consider a system of *N* overdamped Brownian particles in interaction in contact with a heat bath fixing the temperature *T*. Such a dissipative system is described in statistical mechanics by the canonical ensemble. We assume that the binary potential of interaction $u(|r-r^{\prime }|)$ is long-ranged so that a mean field approximation can be implemented when N≫1. We also assume that the particles experience microscopic constraints that affect their dynamics. Under these conditions, the relevant thermodynamical potential is the generalized free energy (see reference [50] for a justification of the generalized free energy functional F[ρ] from a microscopic approach. Note that we have ignored additional constant terms depending on the temperature in the expression of *F*, *E* and *S*; see [50] for more details)

(11) $$\begin{array}{c} F[\rho ]=E[\rho ]-TS[\rho ], \end{array}$$

where

(12) $$\begin{array}{c} E\left[\rho \right]=\frac{1}{2}\int \rho \Phi \;dr=\frac{1}{2}\int \rho \left(r,t\right)u(|r-r^{\prime }\left|\right)\rho \left(r^{\prime },t\right)\;drdr^{\prime } \end{array}$$

is the mean field energy of interaction and

(13) $$\begin{array}{c} S\left[\rho \right]=-\frac{k_{B}}{m}\int C\left(\rho \right)\;dr \end{array}$$

is a generalized entropy which takes into account the microscopic constraints acting on the particles (short-range interactions). Because of these microscopic constraints the entropy *S* may differ from the ideal Boltzmann entropy corresponding to $C\left(\rho \right)=\rho \ln\left(\rho /\rho_{*}\right)$. This leads to a notion of generalized thermodynamics. For reasons that will become clear below we assume that $C^{\prime \prime }>0$ so that C(ρ) is a convex function. The generalized free energy can be written as

(14) $$\begin{array}{c} F\left[\rho \right]=\frac{k_{B}T}{m}\int C\left(\rho \right)\;dr+\frac{1}{2}\int \rho \Phi \;dr \end{array}$$

or, more explicitly, as

(15) $$\begin{array}{c} F\left[\rho \right]=\frac{k_{B}T}{m}\int C\left(\rho \right)\;dr+\frac{1}{2}\int \rho \left(r,t\right)u(|r-r^{\prime }\left|\right)\rho \left(r^{\prime },t\right)\;drdr^{\prime }. \end{array}$$

When the particles are submitted to an external potential $\Phi_{\mathrm{ext}}\left(r\right)$ we must include the contribution of the external energy

(16) $$\begin{array}{c} E_{\mathrm{ext}}\left[\rho \right]=\int \rho \Phi_{\mathrm{ext}}\;dr \end{array}$$

in the expression of the free energy.

### 2.2. Minimum Free Energy Principle

The canonical probability density of the distribution ρ(r) at statistical equilibrium is

(17) $$\begin{array}{c} P_{\mathrm{eq}}\left[\rho \right]=\frac{1}{Z(T)}e^{-F\left[\rho \right]/k_{B}T}\delta \left(M\left[\rho \right]-M\right), \end{array}$$

where

(18) $$\begin{array}{c} Z\left(T\right)=\int e^{-F\left[\rho \right]/k_{B}T}\;D\rho \end{array}$$

is the partition function determined by the normalization condition ∫P[ρ]Dρ=1. The free energy is $F\left(T\right)=-k_{B}T\ln Z\left(T\right)$. In the limit N→+∞, the density ρ(r) of the system is strongly peaked around the “most probable state” which is the global minimum of the free energy F[ρ] at fixed mass M=∫ρdr. Therefore, the equilibrium state of the system in the canonical ensemble is the solution of the minimization problem:

(19) $$\begin{array}{c} \min_{\rho (r)}\;\left\{F\left[\rho \right]\;|\;M\left[\rho \right]=M\right\}. \end{array}$$

This is the canonical version of the maximum entropy principle (Boltzmann principle) that holds in the microcanonical ensemble for isolated systems evolving at fixed mass and energy. For N≫1, the partition function and the free energy can be approximated by

(20) $$\begin{array}{c} Z\left(T\right)≃e^{-F\left[\rho_{*}\right]/k_{B}T}\;\mathrm{and}\;F\left(T\right)≃F\left[\rho_{*}\right], \end{array}$$

where $\rho_{*}\left(r\right)$ is the solution of Equation (19). This formula can be interpreted as a large deviation result.

The extrema of the generalized free energy at fixed mass are determined by the variational principle

(21) $$\begin{array}{c} \delta F-\frac{\mu }{m}\delta M=0, \end{array}$$

where μ is a Lagrange multiplier taking into account the mass constraint. It can be identified with the chemical potential in thermodynamics. The variational principle from Equation (21) leads to the Gibbs relation

(22) $$\begin{array}{c} \frac{\delta F}{\delta \rho }=\frac{\mu }{m}. \end{array}$$

An extremum of free energy at fixed mass ρ(r) is a (local) minimum of free energy at fixed mass if, and only if,

(23) $$\begin{array}{c} \delta^{2}F=\frac{1}{2}\int \frac{\delta^{2}F}{\delta \rho \left(r\right)\delta \rho \left(r^{\prime }\right)}\delta \rho \left(r\right)\delta \rho \left(r^{\prime }\right)\;drdr^{\prime }>0 \end{array}$$

for all perturbations δρ that conserve mass. This is the condition of thermodynamical stability in the canonical ensemble.

The statistical equilibrium state of the system is dominated by the global minimum of free energy at fixed mass. It corresponds to a fully stable state. Local minima of free energy at fixed mass are metastable states. We will see that metastable states may be very relevant for systems with long-range interactions because they have very long lifetimes scaling as $e^{N}$, where *N* is the number of particles in the system [118]. By contrast, maxima or saddle points of free energy at fixed mass are unstable equilibrium states and must be discarded.

### 2.3. Thermodynamical Equilibrium States

For a generalized free energy of the form of Equation (14), the thermodynamical equilibrium states determined by Equation (22) are given by

(24) $$\begin{array}{c} \frac{k_{B}T}{m}C^{\prime }\left(\rho \right)+\Phi =\frac{\mu }{m} \end{array}$$

or, equivalently, by

(25) $$\begin{array}{c} C^{\prime }\left(\rho \right)=-\beta m\Phi +\alpha , \end{array}$$

where

(26) $$\begin{array}{c} \alpha =\frac{\mu }{k_{B}T} \end{array}$$

is a constant. Since *C* is a convex function, this equation can be reversed to give

(27) $$\begin{array}{c} \rho (r)=F(\beta m\Phi (r)-\alpha ), \end{array}$$

where

(28) $$\begin{array}{c} F\left(x\right)={\left(C^{\prime }\right)}^{-1}\left(-x\right) \end{array}$$

is a monotonically decreasing function. Therefore, at statistical equilibrium, the density is a monotonically decreasing function ρ=ρ(Φ) of the potential (we assume T>0). (We note that Equation (27) determines the equilibrium density–potential relation ρ(Φ)=F(βmΦ−α) in terms of the generalized entropy C(ρ) through Equation (28). Inversely, the generalized entropy is determined by the equilibrium density–potential relation through the relation

(29) $$\begin{array}{c} C\left(\rho \right)=-\int^{\rho }F^{-1}\left(x\right)dx, \end{array}$$

where $F^{-1}$ is the reciprocal of *F*.) We have the identity

(30) $$\begin{array}{c} \rho^{\prime }\left(\Phi \right)=-\frac{\beta m}{C^{\prime \prime }\left(\rho \right)}<0. \end{array}$$

Substituting Equation (6) into Equation (25), we find that the equilibrium density ρ(r) is determined by an integral equation of the form

(31) $$\begin{array}{c} C^{\prime }\left[\rho \left(r\right)\right]=-\beta m\int \rho \left(r^{\prime }\right)u(|r-r^{\prime }\left|\right)\;dr^{\prime }+\alpha . \end{array}$$

The Lagrange multiplier α is obtained from the condition ∫ρdr=M. Alternatively, substituting Equation (27) into Equation (6), we find that the equilibrium potential Φ(r) is determined by an equation of the form

(32) $$\begin{array}{c} \Phi \left(r\right)=\int u(|r-r^{\prime }\left|\right)F\left[\beta m\Phi \left(r^{\prime }\right)-\alpha \right]\;dr^{\prime }, \end{array}$$

which is the standard Hammerstein form of nonlinear integral equations.

Bibliographic note: The integral Equation (31) was introduced in full generality in [97]. However, a particular form of this equation associated with the van der Waals equation of state appears in a paper of van Kampen [111].

### 2.4. Thermodynamical Stability

For a generalized free energy of the form of Equation (14), the condition of thermodynamical stability from Equation (23) takes the form

(33) $$\begin{array}{c} \delta^{2}F=\frac{k_{B}T}{m}\int C^{\prime \prime }\left(\rho \right)\frac{{\left(\delta \rho \right)}^{2}}{2}\;dr+\frac{1}{2}\int \delta \rho \delta \Phi \;dr>0 \end{array}$$

for all perturbations δρ that conserve mass. Using Equation (30), the condition of thermodynamical stability can also be written as

(34) $$\begin{array}{c} -\int \frac{{\left(\delta \rho \right)}^{2}}{\rho^{\prime }\left(\Phi \right)}dr+\int \delta \rho \delta \Phi dr>0 \end{array}$$

for all perturbations δρ that conserve mass.

Remark: In the presence of a fixed external potential $\Phi_{\mathrm{ext}}\left(r\right)$, the foregoing equations remain valid provided that Φ is replaced by $\Phi +\Phi_{\mathrm{ext}}$. In the absence of self-interaction (Φ=0), there is a unique equilibrium state given by $\rho \left(r\right)=F(\beta m\Phi_{\mathrm{ext}}\left(r\right)-\alpha )$. Since the second variations of the free energy $\delta^{2}F=-T\delta^{2}S=\left(k_{B}T/2m\right)\int C^{\prime \prime }\left(\rho \right){\left(\delta \rho \right)}^{2}$ are always positive when *C* is convex, this equilibrium state is the global minimum of F[ρ] at fixed mass. Therefore, it is always thermodynamically stable. In the presence of self-interaction, the term $\frac{1}{2}\int \delta \rho \delta \Phi \;dr$ in Equation (33) may be negative so that the stability of the equilibrium state is not granted. It may be either stable or unstable. This demands a specific study depending on the potential of interaction.

## 3. Macroscopic Description: Average Dynamics

### 3.1. Generalized Deterministic Mean Field Smoluchowski Equation

We consider the mean field Smoluchowski Equation (5) but we allow the diffusion coefficient *D* and the mobility χ to depend on the local density ρ(r,t). This leads to the generalized mean field Smoluchowski equation

(35) $$\begin{array}{c} \frac{\partial \rho }{\partial t}=\nabla \cdot \left(Dh(\rho )\nabla \rho +\chi g(\rho )\nabla \Phi \right), \end{array}$$

where the potential Φ(r,t) is given by Equation (6). The generalized mean field Smoluchowski equation can be written more explicitly as

(36) $$\begin{array}{c} \frac{\partial \rho }{\partial t}=\nabla \cdot \left[Dh\left(\rho \right)\nabla \rho +\chi g\left(\rho \right)\nabla \int \rho \left(r^{\prime },t\right)u(|r-r^{\prime }\left|\right)\;dr^{\prime }\right]. \end{array}$$

Here, h(ρ) and g(ρ) are positive functions of the density. These notations are chosen so that the usual Smoluchowski equation with a constant diffusion *D* and a constant mobility χ is recovered for h(ρ)=1 and g(ρ)=ρ. The functions h(ρ) and g(ρ) take into account microscopic constraints (excluded volume effects, steric hindrance, lattice, crowded environments, etc.) that act on the particles at small scales and modify their dynamics. As we shall see, these functions are related to the generalized entropy introduced in Section 2 (or inversely). When the particles are submitted to an external potential $\Phi_{\mathrm{ext}}\left(r\right)$ we must add its contribution in Equation (35) by making the substitution $\Phi \left(r,t\right)\to \Phi \left(r,t\right)+\Phi_{\mathrm{ext}}\left(r\right)$.

The generalized mean field Smoluchowski Equation (35) can be written under the conservative form

(37) $$\begin{array}{c} \frac{\partial \rho }{\partial t}=-\nabla \cdot J, \end{array}$$

where

(38) $$\begin{array}{c} J=-\left(Dh(\rho )\nabla \rho +\chi g(\rho )\nabla \Phi \right) \end{array}$$

is the diffusion current. The structure of Equation (37) expresses the local conservation of mass. It guarantees the conservation of the total mass M=∫ρdr provided that the normal component of the current at the boundary vanishes.

Bibliographic note: The generalized deterministic mean field Smoluchowski Equation (35) was introduced in full generality in [97,102] (see reviews in [50,106]). However, particular forms of this equation (associated with the Fermi–Dirac entropy in configuration space) appeared earlier in the context of 2D turbulence [119,120,121], lattice gases with long-range interactions [21], kinetic theory of adsorbates [25] and bacterial populations undergoing chemotaxis [122]. The generalized deterministic mean field Smoluchowski Equation (35) can be applied to various systems (electrolytes, chemotaxis, superconductors, self-gravitating Brownian particles, 2D vortices, directed self-assembly of nanoparticles, colloids at a fluid interface, nucleation, BMF model, etc.) as discussed in [50,106,123,124,125]. For example, the generalized deterministic mean field Smoluchowski Equation (35) has been studied in the context of self-gravitating Brownian particles and bacterial populations [34,126,127] (it has been studied specifically with the Tsallis entropy in [128,129,130,131] and with the Fermi–Dirac entropy in [102,123,132]), 2D vortices [133,134,135] and colloids at a fluid interface [47]. More general equations taking inertial effects into account are considered in [35,63,97,98,99,100,101,106,136,137].

### 3.2. Generalized Langevin Equation

The generalized mean field Smoluchowski Equation (35) can be rewritten as

(39) $$\begin{array}{c} \frac{\partial \rho }{\partial t}=\nabla \cdot \left[\nabla (D(\rho )\rho )+\chi (\rho )\rho \nabla \Phi \right], \end{array}$$

where D(ρ) is a density-dependent diffusion coefficient and χ(ρ) is a density-dependent mobility defined by

(40) $$\begin{array}{c} Dh\left(\rho \right)=\frac{d}{d\rho }\left[D(\rho )\rho \right],\;\chi \left(\rho \right)=\chi \frac{g(\rho )}{\rho }. \end{array}$$

The generalized mean field Smoluchowski Equation (39) can be obtained from the generalized mean field Langevin equation

(41) $$\begin{array}{c} \frac{dr}{dt}=-\chi \left(\rho \right)\nabla \Phi +\sqrt{2D(\rho )}\;R\left(t\right), \end{array}$$

where R(t) is a Gaussian white noise satisfying 〈R(t)〉=0 and $\langle R_{i}\left(t\right)R_{j}\left(t^{\prime }\right)\rangle =\delta_{ij}\delta \left(t-t^{\prime }\right)$. We note that the noise is multiplicative since it depends on the position r through the function D(ρ(r,t)). To obtain the generalized mean field Smoluchowski Equation (39) from the generalized mean field Langevin Equation (41), we must use the Ito prescription for the noise (if we use the Stratonovich prescription we must account for a spurious drift term).

### 3.3. Generalized Einstein Relation

An equilibrium state of the generalized mean field Smoluchowski Equation (35) satisfies the condition J=0 (a stationary solution $\partial_{t}\rho =0$ of the generalized mean field Smoluchowski equation satisfies the condition ∇·J=0. An equilibrium state satisfies the stronger condition J=0. In this paper, we consider boundary conditions such that ∇·J=0 implies J=0. As a result, a stationary solution is an equilibrium solution), leading to

(42) $$\begin{array}{c} \frac{D}{\chi }\frac{h(\rho )}{g(\rho )}\nabla \rho +\nabla \Phi =0. \end{array}$$

We require that an equilibrium state of the generalized mean field Smoluchowski equation is a thermodynamical equilibrium state as defined in Section 2. Taking the gradient of Equation (25) we get

(43) $$\begin{array}{c} \frac{k_{B}T}{m}C^{\prime \prime }\left(\rho \right)\nabla \rho +\nabla \Phi =0. \end{array}$$

Comparing Equations (42) and (43), we obtain the generalized Einstein relation

(44) $$\begin{array}{c} \frac{D}{\chi }\frac{h(\rho )}{g(\rho )}=\frac{k_{B}T}{m}C^{\prime \prime }\left(\rho \right). \end{array}$$

We can always define the parameters *D* and χ in the generalized mean field Smoluchowski Equation (35) so that the following relations

(45) $$\begin{array}{c} D=\frac{\chi k_{B}T}{m} \end{array}$$

and

(46) $$\begin{array}{c} C^{\prime \prime }\left(\rho \right)=\frac{h(\rho )}{g(\rho )} \end{array}$$

are satisfied individually. In this manner, the ordinary Einstein relation from Equation (2) is preserved in the generalized thermodynamical framework. On the other hand, Equation (46) determines the generalized entropy from Equation (13) in terms of the functions h(ρ) and g(ρ) appearing in the generalized Smoluchowski Equation (35). (Inversely, the generalized entropy C(ρ) does not determine h(ρ) and g(ρ) individually, but only their ratio h(ρ)/g(ρ). This means that we can construct an infinity of generalized Smoluchowski equations associated with the same entropy. For example, the two equations of Section 3.4 are different but they are associated with the same entropy.) For ideal systems for which h(ρ)=1 and g(ρ)=ρ, we find that $C^{\prime \prime }\left(\rho \right)=1/\rho$, returning the Boltzmann entropy $C\left(\rho \right)=\rho \ln\left(\rho /\rho_{*}\right)$. Examples of generalized entropies are given in Appendix A and in [50,106,108,109].

### 3.4. Particular Forms of the Generalized Smoluchowski Equation

If we take h(ρ)=1 and $g\left(\rho \right)=1/C^{\prime \prime }\left(\rho \right)$, the generalized Smoluchowski Equation (35) can be expressed in terms of the generalized entropy under the form

(47) $$\begin{array}{c} \frac{\partial \rho }{\partial t}=\nabla \cdot \left(D\nabla \rho +\frac{\chi }{C^{\prime \prime }\left(\rho \right)}\nabla \Phi \right). \end{array}$$

In that case, we have a constant diffusion D(ρ)=D and a density-dependent mobility $\chi \left(\rho \right)=\chi /(\rho C^{\prime \prime }\left(\rho \right))$.

If we take g(ρ)=ρ and $h\left(\rho \right)=\rho C^{\prime \prime }\left(\rho \right)$, the generalized Smoluchowski Equation (35) can be expressed in terms of the generalized entropy under the form

(48) $$\begin{array}{c} \frac{\partial \rho }{\partial t}=\nabla \cdot \left(D\rho C^{\prime \prime }\left(\rho \right)\nabla \rho +\chi \rho \nabla \Phi \right). \end{array}$$

In that case, we have a constant mobility χ(ρ)=χ and a density-dependent diffusion $D\left(\rho \right)=D\rho {\left[C\left(\rho \right)/\rho \right]}^{\prime }$. Note that the condition D(ρ)≥0 requires that ${\left[C\left(\rho \right)/\rho \right]}^{\prime }\geq 0$ (this condition, which corresponds to a positive pressure – see Appendix A – is not strictly compulsory). This gives a constraint on the possible forms of C(ρ).

### 3.5. Gradient Flow

The connection between the generalized mean field Smoluchowski Equation (35) and the generalized free energy from Equation (14) is made clear by writing the Smoluchowski equation as a gradient flow. Taking the functional derivative of the generalized free energy from Equation (14) with respect to the density, we get

(49) $$\begin{array}{c} \frac{\delta F}{\delta \rho }=\frac{k_{B}T}{m}C^{\prime }\left(\rho \right)+\Phi . \end{array}$$

Its gradient is

(50) $$\begin{array}{c} \nabla \frac{\delta F}{\delta \rho }=\frac{k_{B}T}{m}C^{\prime \prime }\left(\rho \right)\nabla \rho +\nabla \Phi . \end{array}$$

On the other hand, the Smoluchowski current from Equation (38) can be written as

(51) $$\begin{array}{c} J=-\chi g\left(\rho \right)\left(\frac{D}{\chi }\frac{h(\rho )}{g(\rho )}\nabla \rho +\nabla \Phi \right). \end{array}$$

Using the generalized Einstein relation from Equation (44), we get

(52) $$\begin{array}{c} J=-\chi g\left(\rho \right)\left[\frac{k_{B}T}{m}C^{\prime \prime }\left(\rho \right)\nabla \rho +\nabla \Phi \right]. \end{array}$$

Comparing Equations (50) and (52), we observe that

(53) $$\begin{array}{c} J=-\chi g\left(\rho \right)\nabla \frac{\delta F}{\delta \rho }. \end{array}$$

This equation shows that the Smoluchowski current is proportional to the gradient of the functional derivative of the free energy (see Section 3.8). This is called a gradient flow. Substituting Equation (53) into Equation (37), we find that the generalized mean field Smoluchowski equation can be written as

(54) $$\begin{array}{c} \frac{\partial \rho }{\partial t}=\nabla \cdot \left[\chi g\left(\rho \right)\nabla \frac{\delta F}{\delta \rho }\right]. \end{array}$$

This gradient flow formulation is very general. In particular, the properties derived below are valid for an arbitrary form of free energy functional F[ρ] which is not necessarily of the form of Equation (14).

### 3.6. Equilibrium States

An equilibrium state of the gradient flow Equation (54) satisfies the condition J=0, leading to

(55) $$\begin{array}{c} \frac{\delta F}{\delta \rho }=\frac{\mu }{m}, \end{array}$$

where μ is a constant of integration. Comparing Equation (55) with Equation (22), we see that an equilibrium state of the gradient flow equation is an extremum of free energy at fixed mass (thermodynamical equilibrium state). When the free energy is given by Equation (14), Equation (55) reduces to Equation (24) and we can directly check that it is an equilibrium solution of Equation (35) by using the generalized Einstein relation from Equation (44).

### 3.7. H-Theorem

The gradient flow Equation (54) implies that the free energy decreases monotonically with time provided that χg(ρ) is positive. Indeed, the rate of dissipation of free energy is given by

(56) $$\begin{array}{c} \dot{F}=\int \frac{\delta F}{\delta \rho }\frac{\partial \rho }{\partial t}\;dr=-\int \frac{\delta F}{\delta \rho }\nabla \cdot J\;dr=\int J\cdot \nabla \frac{\delta F}{\delta \rho }\;dr. \end{array}$$

To get the last equality, we have integrated by parts and assumed that the normal component of the current vanishes on the boundary. Substituting Equation (53) into Equation (56) we obtain

(57) $$\begin{array}{c} \dot{F}=-\int \frac{J^{2}}{\chi g(\rho )}\;dr\;i.e.\;\dot{F}=-\int \chi g\left(\rho \right){\left(\nabla \frac{\delta F}{\delta \rho }\right)}^{2}\;dr. \end{array}$$

Therefore $\dot{F}\leq 0$ and $\dot{F}=0$ if, and only if, J=0. This is the canonical ensemble version of the *H*-theorem. We have therefore established that

(58) $$\begin{array}{c} \dot{F}\leq 0,\;\dot{F}=0\;\Leftrightarrow \;J=0. \end{array}$$

A functional that satisfies these properties is called a Lyapunov functional. Using Lyapunov’s direct method, one can show that an equilibrium state ρ(r) of Equation (54) is (linearly) dynamically stable if, and only if, it is a (local) minimum of *F* at fixed mass. Maxima and saddle points of *F* at fixed mass are linearly unstable. Furthermore, if *F* is bounded from below, the *H*-theorem implies that the system converges towards a stable equilibrium state for t→+∞. If the free energy possesses several local minima, the choice of the equilibrium state depends on a complicated notion of basin of attraction (for self-gravitating Brownian particles [28,29] the free energy is not bounded from below. In that case, the system can either relax towards a local minimum of free energy *F* at fixed mass – when it exists – or collapse to a Dirac peak [30], leading to a divergence of the free energy F(t)→−∞). According to the preceding results, we conclude that dynamical stability with respect to the generalized mean field Smoluchowski equation and generalized thermodynamical stability in the canonical ensemble coincide.

Remark: As discussed in [106,135] the generalized mean field Smoluchowski Equation (35) can provide a numerical algorithm to construct dynamically stable steady states of the mean field barotropic Euler equation, mean field Kramers equation, Vlasov equation and 2D Euler-Poisson equations. This numerical algorithm may be useful because it is not always easy to compute steady states of these equations and make sure that they are dynamically stable.

### 3.8. Onsager’s Linear Thermodynamics

If we define a time-dependent chemical potential by the relation

(59) $$\begin{array}{c} \frac{\mu (r,t)}{m}\equiv \frac{\delta F}{\delta \rho }, \end{array}$$

we find that μ(r,t) is uniform at equilibrium. Indeed, the Gibbs relation from Equation (22) can be written as

(60) $$\begin{array}{c} \mu (r,t)=\mu \;(\mathrm{equilibrium}). \end{array}$$

On the other hand, the current from Equation (53) associated with the gradient flow Equation (54) can be written as

(61) $$\begin{array}{c} J=-\frac{1}{m}\chi g\left(\rho \right)\nabla \mu \left(r,t\right). \end{array}$$

It is proportional to the gradient of the chemical potential. The coefficient of proportionality is a density-dependent mobility. Since the chemical potential is uniform at equilibrium, the current vanishes: J=0. A linear relation of the form J=−λ∇μ between the current and the chemical potential is the simplest relation that we can imagine in order to have J=0 at equilibrium. Such a linear relationship is expected to be always valid close to equilibrium where the gradient of μ is small. This relation is similar to Ohm’s law for electrical condition, Fourier’s law for heat conduction and Fick’s law for diffusion.

These relations correspond to Onsager’s linear thermodynamics [138,139]. This is a phenomenological approach saying that, close to equilibrium, the currents are proportional to the “thermodynamic forces” that cause them. The thermodynamic forces (or “restoring forces”) are equal to the derivative of the “thermodynamic potential” so that they vanish at equilibrium where the thermodynamic potential is extremum. In the present case, the thermodynamic potential is the free energy *F* and the thermodynamic force is ∇(δF/δρ). This formulation implies the *H*-theorem. Indeed, the rate of free energy is (see Equation (56))

(62) $$\begin{array}{c} \dot{F}=\int J\cdot \nabla \frac{\delta F}{\delta \rho }\;dr. \end{array}$$

If we relate the current J linearly to the thermodynamic force ∇(δF/δρ) so that

(63) $$\begin{array}{c} J=-\chi \left(r,t\right)\nabla \frac{\delta F}{\delta \rho }, \end{array}$$

we get the *H*-theorem $\dot{F}\leq 0$ provided that χ>0. Note that Onsager’s linear thermodynamics does not give the expression of χ(r,t). In the present situation, Equation (53) is consistent with Onsager’s linear thermodynamics with χ(r,t)=χg(ρ). For a free energy F[ρ] of the form of Equation (14), Onsager’s linear thermodynamics yields the generalized Smoluchowski Equation (35). This shows that Onsager’s linear thermodynamics can be valid even if we are far from equilibrium since there is no restriction on the validity of the Smoluchowski equation.

### 3.9. Maximum Free Energy Dissipation Principle

The current from Equation (53) can be obtained from a variational principle called the maximum free energy dissipation principle. At equilibrium, the density ρ(r) minimizes the free energy F[ρ] at fixed mass *M* (see Section 2). This minimum free energy principle is the canonical ensemble version of the maximum entropy principle introduced by Boltzmann. It characterizes the most probable state of a system at statistical equilibrium. In a similar manner, out-of-equilibrium, the current J maximizes the rate of free energy dissipation $-\dot{F}\left[J\right]-E_{d}\left[J\right]$. This maximum free energy dissipation principle is the canonical ensemble version of the maximum entropy production principle (for isolated systems described by the microcanonical ensemble, the increase of entropy during the evolution must be the highest while conserving the energy: $\dot{S}$ maximum at fixed energy. For dissipative systems described by the canonical ensemble, the decrease of free energy during the evolution must be the highest: -$\dot{F}$ maximum). It describes the most probable course of an irreversible process. It can be viewed as a variational formulation of Onsager’s linear thermodynamics (see Section 3.8).

The rate of dissipation of free energy is given by (see Equation (56))

(64) $$\begin{array}{c} \dot{F}=\int J\cdot \nabla \frac{\delta F}{\delta \rho }\;dr. \end{array}$$

We introduce the dissipation function

(65) $$\begin{array}{c} E_{d}=\int \frac{J^{2}}{2\chi g(\rho )}\;dr. \end{array}$$

Let us determine the current J which maximizes the rate of free energy dissipation $-\dot{F}\left[J\right]-E_{d}\left[J\right]$ with the convention that the density ρ(r,t) is prescribed. Therefore, we consider the maximization problem

(66) $$\begin{array}{c} \max_{J}\left\{-\dot{F}\left[J\right]-E_{d}\left[J\right]\right\}. \end{array}$$

The extrema of free energy dissipation are determined by the variational principle

(67) $$\begin{array}{c} \delta (-\dot{F}-E_{d})=0. \end{array}$$

There is a single extremum given by

(68) $$\begin{array}{c} J=-\chi g\left(\rho \right)\nabla \frac{\delta F}{\delta \rho }, \end{array}$$

corresponding to the Smoluchowski current from Equation (53). Since

(69) $$\begin{array}{c} \delta^{2}\left(-\dot{F}-E_{d}\right)=-\int \frac{{\left(\delta J\right)}^{2}}{2\chi g(\rho )}dr\leq 0, \end{array}$$

we find that the Smoluchowski current from Equation (68) maximizes the dissipation of free energy. Using Equation (68) we obtain the relation

(70) $$\begin{array}{c} \dot{F}=-2E_{d}, \end{array}$$

which is equivalent to Equation (57). Therefore, the dissipation function equals half the rate of dissipation of free energy. We also introduce the dual dissipation function

(71) $$\begin{array}{c} E_{d}^{*}=\frac{1}{2}\int \chi g\left(\rho \right){\left(\nabla \frac{\delta F}{\delta \rho }\right)}^{2}\;dr. \end{array}$$

We note that $E_{d}^{*}\left[\rho \right]$ is a functional of the density ρ while $E_{d}\left[J\right]$ is a functional of the current J. Using Equation (68) we obtain the relation

(72) $$\begin{array}{c} \dot{F}=-2E_{d}=-2E_{d}^{*}. \end{array}$$

Bibliographic note: the maximum entropy production principle was introduced by Onsager [138,139] to derive his famous reciprocal relations in irreversible processes. It finds its origin in the work of Lord Rayleigh [140,141]. It was used in the context of 2D turbulence and stellar dynamics to derive relaxation equations associated with the theory of violent relaxation [119,120,133,134,135]. It was also used in the context of generalized thermodynamics to derive generalized Fokker-Planck equations [97,102,106].

### 3.10. Equivalence between Dynamical and Thermodynamical Stability

In this section, we derive a simple relation that establishes a direct equivalence between dynamical and thermodynamical stability for the generalized Smoluchowski equation. Let us consider a small perturbation δρ around an equilibrium state of the generalized Smoluchowski Equation (54). We write the time dependence of the perturbation as $\delta \rho ∼e^{\lambda t}$. The perturbation is damped exponentially when λ<0 (stable equilibrium) or increases exponentially when λ>0 (unstable equilibrium). The linearized Smoluchowski equation can be written as

(73) $$\begin{array}{c} \lambda \delta \rho =-\nabla \cdot \delta J \end{array}$$

with

(74) $$\begin{array}{c} \delta J=-\chi g\left(\rho \right)\nabla \int \frac{\delta^{2}F}{\delta \rho \left(r\right)\delta \rho \left(r^{\prime }\right)}\delta \rho \left(r^{\prime },t\right)\;dr^{\prime }. \end{array}$$

The second variations of free energy are given by

(75) $$\begin{array}{c} \delta^{2}F=\frac{1}{2}\int \frac{\delta^{2}F}{\delta \rho \left(r\right)\delta \rho \left(r^{\prime }\right)}\delta \rho \left(r,t\right)\delta \rho \left(r^{\prime },t\right)\;drdr^{\prime }. \end{array}$$

Substituting Equation (73) into Equation (75) we get

(76) $$\begin{array}{c} \lambda \delta^{2}F=-\frac{1}{2}\int drdr^{\prime }\;\frac{\delta^{2}F}{\delta \rho \left(r\right)\delta \rho \left(r^{\prime }\right)}\delta \rho \left(r^{\prime },t\right)\nabla \cdot \delta J. \end{array}$$

Integrating by parts, we find that

(77) $$\begin{array}{c} \lambda \delta^{2}F=\frac{1}{2}\int drdr^{\prime }\;\delta J\cdot \nabla \frac{\delta^{2}F}{\delta \rho \left(r\right)\delta \rho \left(r^{\prime }\right)}\delta \rho \left(r^{\prime },t\right)=-\frac{1}{2}\int \frac{{\left(\delta J\right)}^{2}}{\chi g(\rho )}\;dr, \end{array}$$

where we have used Equation (74) to obtain the second equality. On the other hand, the second variations of the rate of free energy dissipation from Equation (57) around the equilibrium are

(78) $$\begin{array}{c} \delta^{2}\dot{F}=-\int \frac{{\left(\delta J\right)}^{2}}{\chi g(\rho )}\;dr, \end{array}$$

and they are clearly negative. Combining Equations (77) and (78), we finally obtain the following relation (this relation was first obtained in Appendix B of [106] for a free energy of the form of Equation (15). The present calculation extends its domain of validity to an arbitrary functional F[ρ].)

(79) $$\begin{array}{c} 2\lambda \delta^{2}F=\delta^{2}\dot{F}\leq 0. \end{array}$$

This relation shows that a steady state of the generalized Smoluchowski Equation (54) is linearly dynamically stable (λ<0) if, and only if, it is a (local) minimum of free energy at fixed mass ($\delta^{2}F>0$). Therefore, dynamical and generalized thermodynamical stability coincide.

Remark: If we repeat the above calculation by using

(80) $$\begin{array}{c} \frac{\partial \delta \rho }{\partial t}=-\nabla \cdot \delta J \end{array}$$

instead of Equation (73), we obtain

(81) $$\begin{array}{c} \frac{d}{dt}\left(\delta^{2}F\right)=\delta^{2}\dot{F} \end{array}$$

instead of Equation (79). On the other hand, if we take the derivative of $\delta^{2}F$ and use $\partial_{t}\delta \rho =\lambda \delta \rho$, we get

(82) $$\begin{array}{c} \frac{d}{dt}\left(\delta^{2}F\right)=2\lambda \delta^{2}F. \end{array}$$

From Equations (81) and (82) we recover Equation (79).

## 4. Mesoscopic Description: Stochastic Dynamics

### 4.1. Generalized Stochastic Mean Field Smoluchowski Equation

The generalized mean field Smoluchowski Equation (54) is a deterministic equation that ignores fluctuations. It describes the macroscopic evolution of the system through the ensemble average density ρ(r,t). In practice, there are fluctuations around the average dynamics. They arise from the thermal noise and from finite *N* effects. In reference [50] (see also [14]), we have derived a generalized stochastic Smoluchowski equation from the theory of fluctuations developed by Landau and Lifshitz (see [69], chapter XVII). It describes the mesoscopic evolution of the system through the coarse-grained density $\bar{\rho }\left(r,t\right)$ (in the following, we drop the bar on ρ to simplify the notations). It can be written as

(83) $$\begin{array}{c} \frac{\partial \rho }{\partial t}=\nabla \cdot \left[\chi g\left(\rho \right)\nabla \frac{\delta F}{\delta \rho }\right]+\nabla \cdot \left[\sqrt{2\chi k_{B}Tg\left(\rho \right)}\;R\left(r,t\right)\right], \end{array}$$

where R(r,t) is a Gaussian white noise satisfying 〈R(r,t)〉=0 and $\langle R_{i}\left(r,t\right)R_{j}\left(r^{\prime },t^{\prime }\right)\rangle =\delta_{ij}\delta \left(r-r^{\prime }\right)\delta \left(t-t^{\prime }\right)$. We note that, in general, the noise term is multiplicative since it depends on the density through the function g(ρ). This is the case, in particular, for ideal systems for which g(ρ)=ρ. On the other hand, the function h(ρ) does not appear in the noise term. (The noise term is independent of ρ when g(ρ)=1. For h(ρ)=ρ, corresponding to a normal diffusion, the condition g(ρ)=1 leads to $C\left(\rho \right)=\rho^{2}/2$, i.e., $S=-\frac{1}{2}\int \rho^{2}\;dr$. This is a Tsallis entropy of index γ=2.) We also note that Equation (83) relies on a mean field approximation, valid for N≫1, since it involves the mean field free energy from Equation (15). However, the noise keeps track of finite *N* effects since it scales as $1/\sqrt{N}$ after a proper normalization (see Appendix A of [70] and Appendix B of [63]).

The generalized stochastic Smoluchowski Equation (83) can be interpreted as a stochastic Langevin equation for the density ρ(r,t). The corresponding Fokker-Planck equation for the probability density P[ρ,t] of observing the density ρ(r,t) at time *t* is

(84) $$\begin{array}{c} \frac{\partial P}{\partial t}\left[\rho ,t\right]=-\int dr\;\frac{\delta }{\delta \rho (r)}\left\{\nabla \cdot \chi g\left(\rho \right)\nabla \left[k_{B}T\frac{\delta }{\delta \rho (r)}+\frac{\delta F}{\delta \rho (r)}\right]P\left[\rho ,t\right]\right\}. \end{array}$$

This functional Fokker-Planck equation relaxes towards the canonical distribution from Equation (17) provided that we use the Ito prescription for the noise (see Appendix B). Actually, the form of the noise can be determined precisely in order to recover the canonical distribution at equilibrium. (As discussed in Section 2, we are considering a notion of “generalized thermodynamics” in the sense that the entropy S[ρ] may be different from the ideal Boltzmann entropy $S\left[\rho \right]=\rho \ln\left(\rho /\rho_{*}\right)$. However, we still assume that the equilibrium distribution $P_{\mathrm{eq}}\left[\rho \right]$ is given by an exponential (Boltzmann) function of the free energy as in Equation (17). A true generalization of thermodynamics would be obtained by replacing the Boltzmann-Gibbs distribution $P_{\mathrm{eq}}\left[\rho \right]\propto e^{-\beta F[\rho ]}$ by a *q*-distribution $P_{\mathrm{eq}}\left[\rho \right]\propto e_{q}^{-\beta F[\rho ]}$ (Tsallis), or an even more general distribution. This would amount to changing the noise term in Equation (83) by allowing it to depend on P[ρ,t].)

Remark: The stochastic mean field Smoluchowski Equation (83) introduced in [14,50] is fundamentally different from Dean’s equation [77] (see Appendix C). The Dean Equation (A96) is an exact equation satisfied by the discrete density $\rho_{d}\left(r,t\right)=\sum_{i}m\delta \left(r-r_{i}\left(t\right)\right)$ which is a sum of Dirac distributions. It involves the exact free energy $F_{d}=\frac{k_{B}T}{m}\int \rho_{d}\left(\ln\rho_{d}-1\right)\;dr+\frac{1}{2}\int \rho_{d}\left(r,t\right)u(|r-r^{\prime }\left|\right)\rho_{d}\left(r^{\prime },t\right)\;drdr^{\prime }$ constructed with the discrete density $\rho_{d}\left(r,t\right)$. It is equivalent to the *N*-body stochastic Langevin Equation (A77) or to the *N*-body Smoluchowski Equation (A78), describing a system of Brownian particles in interaction and, as such, it contains the complete information on the dynamics of the particles (similarly to the Liouville equation or to the Klimontovich equation for Hamiltonian systems of particles in interaction). It is not clear whether the Dean equation is well-defined mathematically (because of the presence of δ-functions). Furthermore, on a physical point of view, it is not clear whether this “exact” equation can be used on an operational basis. Indeed, since it is equivalent to the *N*-body stochastic Langevin equations, it contains “too much” information on the system. In this sense, it is of little use since it encodes the position of all particles; it is simpler to follow the particle trajectories directly by integrating the Langevin Equation (A77). By contrast, the stochastic mean field Smoluchowski Equation (83) applies to a coarse-grained (smooth) density $\bar{\rho }\left(r,t\right)$ which is more easy to handle in explicit calculations. For ideal systems with long-range interactions, it involves the mean field free energy $F=\frac{k_{B}T}{m}\int \bar{\rho }\left(\ln\bar{\rho }-1\right)\;dr+\frac{1}{2}\int \bar{\rho }\left(r,t\right)u(|r-r^{\prime }\left|\right)\bar{\rho }\left(r^{\prime },t\right)\;drdr^{\prime }$ constructed with the coarse-grained density $\bar{\rho }\left(r,t\right)$. Many authors refer to the Dean equation while they actually consider the stochastic mean field Smoluchowski Equation (83)—or its counterpart Equation (A100) in the theory of simple liquids (see Appendix C)—which describes a coarse-grained mesoscopic dynamics (see Appendix B of [63] for a more complete discussion). This mesoscopic equation can be obtained from the Landau-Lifshitz theory of fluctuating hydrodynamics as shown in [14,50]. On the other hand, for “complex” systems (described by a generalized entropy) for which g(ρ)≠ρ, it does not seem feasible to derive a stochastic equation of the form of Equation (83) directly from Dean’s microscopic approach while this remains possible from the theory of fluctuating hydrodynamics [50]. This clearly shows that the generalized stochastic mean field Smoluchowski Equation (83) is fundamentally different from the Dean equation.

Bibliographic note: The generalized stochastic mean field Smoluchowski Equation (83) was introduced in full generality in [50]. However, a particular form of this equation (associated with the Fermi–Dirac entropy in configuration space) appeared earlier in the context of the kinetic theory of adsorbates [25]. The generalized stochastic mean field Smoluchowski Equation (83) can be applied to other systems (electrolytes, chemotaxis, superconductors, self-gravitating Brownian particles, 2D vortices, directed self-assembly of nanoparticles, colloids at a fluid interface, nucleation, BMF model, etc.) as discussed in [50]. More general equations taking inertial effects into account are considered in [63].

### 4.2. Derivation of the Kramers Formula from the Instanton Theory

In this section, we calculate the escape rate Γ of a system of Brownian particles in interaction across a barrier of free energy by using the instanton theory. This provides a justification of the Kramers formula giving the typical lifetime of a metastable state. We assume that the free energy functional F[ρ] has a local minimum $\rho_{M}\left(r\right)$ (metastable state) and a global minimum $\rho_{S}\left(r\right)$ (stable state) separated by a maximum or a saddle point $\rho_{U}\left(r\right)$ (unstable state). We have seen that Equation (83) can be interpreted as a Langevin equation. In the absence of noise, the evolution is deterministic and the density relaxes to one of the minima of the potential as implied by the *H*-theorem (see Section 3.7). In the presence of noise, the density switches back and forth between the two minima (attractors). When the noise is weak (the weak noise limit corresponds to T→0 and/or N→+∞ [70]), the transition between the two minima is a rare event. One important problem is to determine the rate Γ for the density profile, initially located in the metastable state $\rho_{M}\left(r\right)$, to cross the barrier of free energy and reach the stable state $\rho_{S}\left(r\right)$.

The probability of the path ρ(r,t) is

(85) $$\begin{array}{c} P\left[\rho \left(r,t\right)\right]\propto e^{-S\left[\rho \left(r,t\right)\right]/k_{B}T}, \end{array}$$

where *S* is a generalized Onsager-Machlup functional [142]. In the weak noise limit, using Equation (83), it is given by

(86) $$\begin{array}{c} S\left[\rho \left(r,t\right)\right]=-\frac{1}{4}\int dt\int dr\;\left(\frac{\partial \rho }{\partial t}-\nabla \cdot \left[\chi g\left(\rho \right)\nabla \frac{\delta F}{\delta \rho }\right]\right){\left(\nabla \chi g\left(\rho \right)\nabla \right)}^{-1}\left(\frac{\partial \rho }{\partial t}-\nabla \cdot \left[\chi g\left(\rho \right)\nabla \frac{\delta F}{\delta \rho }\right]\right). \end{array}$$

The functional *S* may be called an action by analogy with the path-integrals formulation of quantum mechanics (the temperature *T* plays the role of the Planck constant *ℏ* in quantum mechanics) [143]. It can be written as S=∫Ldt where *L* is the corresponding Lagrangian. The probability density to observe the system with the profile $\rho_{2}\left(r\right)$ at time $t_{2}$ given that it had the profile $\rho_{1}\left(r\right)$ at time $t_{1}$ is

(87) $$\begin{array}{c} P\left[\rho_{2}\left(r\right),t_{2}|\rho_{1}\left(r\right),t_{1}\right]=\int D\rho \;e^{-S\left[\rho \right]/k_{B}T}, \end{array}$$

where the integral runs over all paths satisfying $\rho \left(r,t_{1}\right)=\rho_{1}\left(r\right)$ and $\rho \left(r,t_{2}\right)=\rho_{2}\left(r\right)$. For a given initial condition $\rho_{0}\left(r\right)$ at $t=t_{0}$, the probability density $P\left[\rho \left(r\right),t\right]\equiv P\left[\rho \left(r\right),t|\rho_{0}\left(r\right),t_{0}\right]$ to observe the system with the profile ρ(r) at time *t* satisfies the functional Fokker-Planck Equation (84). In the weak noise limit, the typical paths explored by the system are concentrated close to the most probable path. In that case, a steepest-descent evaluation of the path integral is possible. The path integral is dominated by the most probable path. To determine the most probable path, we have to minimize the Onsager-Machlup functional S[ρ(r,t)], i.e., we have to solve the minimization problem

(88) $$\begin{array}{c} \min_{\rho (r,t)}\;\left\{S\left[\rho \left(r,t\right)\right]\right\}. \end{array}$$

The equation for the most probable path $\rho_{c}\left(r,t\right)$ that connects two attractors is called an “instanton” [144]. It is obtained by cancelling the first order variations of the action:

(89) $$\begin{array}{c} \delta S=0. \end{array}$$

In the weak noise limit, the transition probability from one state to the other is dominated by the most probable path:

(90) $$\begin{array}{c} P\left[\rho_{2}\left(r\right),t_{2}|\rho_{1}\left(r\right),t_{1}\right]≃e^{-S\left[\rho_{c}\right]/k_{B}T}. \end{array}$$

This formula can be interpreted as a large deviation result (we note the analogies between Equations (85), (87), (88) and (90) and Equations (17)–(20)). It provides an approximate solution of the functional Fokker-Planck Equation (84). On the other hand, it can be shown that the escape rate of the system over the barrier of free energy is given by

(91) $$\begin{array}{c} \Gamma \propto e^{-S\left[\rho_{c}\right]/k_{B}T}, \end{array}$$

where $S[\rho_{c}]$ is the action of the most probable path (instanton) that connects the metastable state to the stable state. In the limit of weak noise, and for a stochastic process that obeys a fluctuation–dissipation relation, it can be shown that the most probable path between the metastable state and the stable state must necessarily pass through the saddle point (playing the role of a “critical droplet” in problems of nucleation). Once the system reaches the saddle point it may either return to the initial metastable state or reach the stable state. In the latter case, it has crossed the barrier of free energy. One can show that the instanton satisfies the equation

(92) $$\begin{array}{c} \frac{\partial \rho }{\partial t}=\pm \nabla \cdot \left[\chi g\left(\rho \right)\nabla \frac{\delta F}{\delta \rho }\right] \end{array}$$

with the boundary conditions $\rho \left(r,-\infty \right)=\rho_{M}\left(r\right)$ and $\rho \left(r,+\infty \right)=\rho_{S}\left(r\right)$. We note that the most probable path corresponds to the deterministic gradient driven dynamics, Equation (54), with a sign ± (considering the solution with the sign +, which corresponds to the downhill solution – see below – we see that the most probable path coincides with the ensemble average path). The physical interpretation of Equation (92) is the following. Starting from the metastable state, the most probable path follows the time-reversed dynamics against the free energy gradient up to the saddle point; beyond the saddle point, it follows the forward-time dynamics down to the stable state. According to Equations (86) and (92), the action of the most probable path corresponding to the transition from the saddle point to the stable state (downhill solution corresponding to Equation (92) with the sign +) is zero while the action of the most probable path corresponding to the transition from the metastable state to the saddle point (uphill solution corresponding to Equation (92) with the sign −) is non zero. This is to be expected since the descent from the saddle point to the stable state is a “free” descent that does not require thermal noise; it thus gives the smallest possible value of zero of the action. By contrast, the rise from the metastable state to the saddle point is a rare event that requires thermal noise. The action for the uphill solution is

(93) $$\begin{array}{c} \begin{array}{ccc} S[\rho (r,t)] & = & \int dt\int dr\;\frac{\partial \rho }{\partial t}{\left(\nabla \chi g\left(\rho \right)\nabla \right)}^{-1}\left(\nabla \cdot \left[\chi g\left(\rho \right)\nabla \frac{\delta F}{\delta \rho }\right]\right)\\ & = & \int dt\int dr\;\frac{\partial \rho }{\partial t}\frac{\delta F}{\delta \rho }\\ & = & \int dt\;\frac{dF}{dt}=\Delta F, \end{array} \end{array}$$

where $\Delta F=F\left[\rho_{U}\right]-F\left[\rho_{M}\right]$ is the barrier of free energy between the metastable state and the unstable state. The total action for the most probable path connecting the attractors is therefore $S_{c}=S\left[\rho_{c}^{-}\right]+S\left[\rho_{c}^{+}\right]=\Delta F+0=\Delta F$. It is determined solely by the uphill path. The instanton solution gives the dominant contribution to the transition rate for a weak noise. Therefore, the rate for the system to pass from the metastable state to the stable state (escape rate) is

(94) $$\begin{array}{c} \Gamma \propto e^{-\Delta F/k_{B}T}. \end{array}$$

This is the celebrated Arrhenius (or Kramers) formula stating that the transition rate is inversely proportional to the exponential of the barrier of free energy divided by $k_{B}T$. (This formula can be simply obtained as follows. The probability of observing the density ρ(r) is $\propto e^{-\beta F[\rho ]}$. Therefore, the probability for the system initially prepared in the metastable state to form a “critical droplet” (unstable state $\rho_{U}$) and then reach the stable state $\rho_{S}$ is $\propto e^{-\beta (F\left[\rho_{U}\right]-F\left[\rho_{M}\right])}$. The typical lifetime of a metastable state may then be estimated by $t_{\mathrm{life}}∼e^{\beta \Delta F}$, where $\Delta F=F\left[\rho_{U}\right]-F\left[\rho_{M}\right]$ is the barrier of free energy between the metastable state and the unstable state.) The typical lifetime of a metastable state is $t_{\mathrm{life}}∼\Gamma^{-1}$. For systems with long-range interactions, the free energy scales as *N* so the typical lifetime of a metastable state scales as

(95) $$\begin{array}{c} t_{\mathrm{life}}\propto e^{N\Delta f/k_{B}T}. \end{array}$$

Therefore, for systems with long-range interactions, the metastable states are very relevant since their lifetime scales as $e^{N}$ with N≫1 [70,118]. Therefore, metastable states are stable in practice. Only very close to the critical point where Δf→0 does their lifetime decrease substantially.

Bibliographic note: A general path-integrals formalism determining the escape rate of a particle moving in a potential V(x) in the weak noise limit has been developed by Bray et al. [145]. Their theory accounts for white noises for which $S[x_{c}]=\Delta V$ (Kramers) and for exponentially correlated noises for which $S[x_{c}]\neq \Delta V$. The instanton theory has been formalized by Freidlin and Wetzel [146] in relation to the theory of large deviations [147]. It has been applied to various systems such as scalar fields described by the Ginzburg-Landau equation [145,148], interacting magnetic moments [149], nucleation [48], two-dimensional fluid flows [150] and Brownian particles with long-range interactions [70].

### 4.3. Relation to the Principle of Maximum Dissipation of Free Energy

It is interesting to discuss the relation between the instanton theory (see Section 4.2) and the principle of maximum dissipation of free energy (see Section 3.9). The Onsager-Machlup functional defined by Equation (86) can be expanded under the form

(96) $$\begin{array}{c} S=\frac{1}{2}\int \left(E_{d}+E_{d}^{*}+\dot{F}\right)\;dt, \end{array}$$

where

(97) $$\begin{array}{c} \begin{array}{ccc} E_{d} & = & \frac{1}{2}\int \frac{\partial \rho }{\partial t}{\left(\nabla \chi g\left(\rho \right)\nabla \right)}^{-1}\frac{\partial \rho }{\partial t}\;dr\\ & = & \frac{1}{2}\int \nabla \cdot J{\left(\nabla \chi g\left(\rho \right)\nabla \right)}^{-1}\nabla \cdot J\;dr\\ & = & \frac{1}{2}\int \frac{J^{2}}{\chi g(\rho )}\;dr, \end{array} \end{array}$$

(98) $$\begin{array}{c} \begin{array}{ccc} E_{d}^{*} & = & \frac{1}{2}\int \nabla \cdot \left[\chi g\left(\rho \right)\nabla \frac{\delta F}{\delta \rho }\right]{\left(\nabla \chi g\left(\rho \right)\nabla \right)}^{-1}\nabla \cdot \left[\chi g\left(\rho \right)\nabla \frac{\delta F}{\delta \rho }\right]\;dr\\ & = & \frac{1}{2}\int \chi g\left(\rho \right){\left(\nabla \frac{\delta F}{\delta \rho }\right)}^{2}\;dr, \end{array} \end{array}$$

and

(99) $$\begin{array}{c} \begin{array}{ccc} \dot{F} & = & -\frac{1}{2}\int \frac{\partial \rho }{\partial t}{\left(\nabla \chi g\left(\rho \right)\nabla \right)}^{-1}\nabla \cdot \left[\chi g\left(\rho \right)\nabla \frac{\delta F}{\delta \rho }\right]\;dr\\ & - & \frac{1}{2}\int \nabla \cdot \left[\chi g\left(\rho \right)\nabla \frac{\delta F}{\delta \rho }\right]{\left(\nabla \chi g\left(\rho \right)\nabla \right)}^{-1}\frac{\partial \rho }{\partial t}\;dr\\ & = & \int \frac{\partial \rho }{\partial t}\frac{\delta F}{\delta \rho }\;dr\\ & = & -\int \nabla \cdot J\frac{\delta F}{\delta \rho }\;dr\\ & = & \int J\cdot \nabla \frac{\delta F}{\delta \rho }\;dr \end{array} \end{array}$$

are the dissipative functions and the rate of free energy introduced in Section 3.9. Equation (96) is the counterpart of Equation (4–18) of Onsager and Machlup [142]. The minimization of the Onsager-Machlup functional *S* with respect to variations on J (at fixed ρ) is therefore equivalent to the maximization of $-\dot{F}-E_{d}$. This returns the principle of maximum dissipation of free energy of Section 3.9 leading to the deterministic Smoluchowski Equation (54). This corresponds to the downhill instanton solution for which we have $\dot{F}=-2E_{d}=-2E_{d}^{*}$ and S=0. On the other hand, for the uphill instanton solution corresponding to the deterministic Smoluchowski Equation (54) with the opposite sign, we have $\dot{F}=2E_{d}=2E_{d}^{*}$ and $S=\int \dot{F}\;dt=\Delta F$.

### 4.4. Boltzmann and Onsager-Machlup Principles

Let us recapitulate the different variational principles considered in this paper. We have seen in Section 2.2 that the probability of the density ρ(r) at statistical equilibrium is given by

(100) $$\begin{array}{c} P_{\mathrm{eq}}\left[\rho \left(r\right)\right]\propto e^{-\beta F[\rho (r)]}\;\left(\mathrm{Boltzmann}\right), \end{array}$$

where F[ρ(r)] is the free energy functional. On the other hand, we have seen in Section 4.2 that, out-of-equilibrium, the probability of the path ρ(r,t) is given by

(101) $$\begin{array}{c} P\left[\rho \left(r,t\right)\right]\propto e^{-\beta S[\rho (r,t)]}\;\left(\mathrm{Onsager}-\mathrm{Machlup}\right), \end{array}$$

where S[ρ(r,t)] is the Onsager-Machlup functional (action) which can be expressed in terms of the rate of free energy and dissipation functions (see Section 4.3). The Onsager-Machlup theorem, Equation (101), is analogous to the Boltzmann principle, Equation (100). (The Boltzmann formula – which was actually formulated by Planck – is usually expressed in the form $S=k_{B}\ln W$, where *W* is the number of complexions. The reversed expression $W\propto e^{S/k_{B}}$, interpreted as the probability of a fluctuation, was formulated by Einstein [151].) The Boltzmann principle tells the probability of an equilibrium state in terms of its entropy (or free energy in the canonical ensemble); the Onsager-Machlup theorem tells the probability of a temporal succession of states in terms of the rate of entropy (or free energy in the canonical ensemble) and dissipation functions. The most probable equilibrium state ρ(r) is obtained by minimizing the free energy F[ρ] at fixed mass. This leads to the generalized Boltzmann distribution from Equation (22). The most probable path is obtained by minimizing the Onsager-Machlup functional *S* or equivalently by maximizing the rate of production of free energy $-\dot{F}-E_{d}$ with respect to variations on J (at fixed ρ). This leads to the generalized deterministic Smoluchowski Equation (54). Therefore, the Onsager-Machlup principle can be viewed as the out-of-equilibrium version of the Boltzmann principle of statistical equilibrium.

## 5. Conclusions

In this paper, we have discussed the main properties of the generalized stochastic Smoluchowski Equation (83). This equation describes the mesoscopic dynamics of a system of overdamped Brownian particles in interaction. For short times, we can neglect the fluctuations and use the deterministic Smoluchowski Equation (54). In that case, the system relaxes towards a stable equilibrium state which is a (local) minimum of free energy at fixed mass. On longer times, or close to the critical point, the fluctuations have to be taken into account. If the equilibrium state achieved by the system is metastable (local but not global minimum of free energy), the fluctuations can allow the system to overcome a barrier of free energy and reach the fully stable state. More generally, if the system possesses several equilibrium states, the fluctuations can allow it to switch between these different equilibrium states and explore the free energy landscape. Such random transitions have been illustrated numerically by Chavanis and Delfini [70] for a model of self-gravitating Brownian particles and bacterial populations presenting two symmetric metastable states. They are expected to be generic of many other systems. We have derived the Kramers formula giving the typical lifetime of a metastable state from the theory of instantons and we have mentioned that this lifetime is extremely long for systems with long-range interactions as is scale as $e^{N}$ [118]. A transition from an equilibrium state to another is therefore a rare event.

In our approach, we have assumed that the Brownian particles experience long-range and short-range interactions. The long-range interactions have been treated in a mean field approximation which becomes exact when N→+∞. The short-range interactions have been treated heuristically with the help of a generalized entropy C(ρ). For “ideal” systems without short-range interactions, the correct entropy is the Boltzmann entropy. It can be obtained from a combinatorial analysis assuming that all the accessible microstates are equiprobable. For “complex” systems, the short-range interactions and the correlations between the particles may forbid some microstates and, therefore, modify the form of the entropy. This type of arguments leads, for example, to the van der Waals entropy given by Equation (A24) [110,111] or to the Fermi–Dirac entropy in position space given by Equation (A29) [81,82,102,132], which take into account excluded volume effects. More generally, the microscopic constraints can generate various forms of entropies characterized by a convex function C(ρ) [50].

We have shown in Appendix C that the generalized entropy C(ρ) appropriate to a given system can be derived in principle from the density functional theory developed in the physics of simple liquids [152]. In that case, it corresponds to the so-called “excess entropy” which arises from the development of correlations due to short-range interactions. In many models of liquids, the correlations can be taken into account by replacing the bare potential of interaction $u(|r-r^{\prime }|)$ by an effective potential of interaction $u_{\mathrm{eff}}(|r-r^{\prime }\left|\right)=-\frac{k_{B}T}{m^{2}}c(|r-r^{\prime }\left|\right)$ proportional to the direct correlation function $c(|r-r^{\prime }|)$. Many methods have been developed in the physics of simple liquids to determine the direct correlation function and the excess free energy for a given potential of short-range interactions [152].

The density functional theory developed in the physics of simple liquids allows us to demystify the notion of “generalized” thermodynamics [109] by showing that “generalized” entropies can be obtained from first principles. They are determined by the potential of short-range interactions $u_{\mathrm{SR}}(|r-r^{\prime }\left|\right)$ in a nontrivial manner explained in [152]. In that sense, what we call “generalized” thermodynamics is just “ordinary” thermodynamics with a “generalized” (excess) form of entropy taking into account microscopic constraints and correlations among the particles. (This correspondance between generalized thermodynamics [109] and density functional theory [152] is obtained when the microscopic constraints act in configuration space as in [50], so that the velocity distribution function remains maxwellian. When the microscopic constraints act in phase space as in [97], implying non-maxwellian velocity distributions, the situation is more complicated. The passage from the generalized Kramers equation to the generalized Smoluchowski equation in the strong friction limit ξ→+∞ is treated in detail in [100,106].)

We hope that one virtue of our paper is to have made a first connection between different communities, generalized thermodynamics [108,109], systems with long-range interactions [12,13] and the physics of simple liquids [152], by showing that their methods are complementary to each other. On the other hand, many results can be extended to the quantum regime as shown in recent papers (see, e.g., [3] and references therein).

## Appendix A. The Generalized Pressure

### Appendix A.1. Equation of State

In this Appendix, we take g(ρ)=ρ (constant mobility). In that case, the generalized mean field Smoluchowski Equation (35) can be written as

(A1) $$\begin{array}{c} \xi \frac{\partial \rho }{\partial t}=\nabla \cdot \left[\frac{k_{B}T}{m}\rho C^{\prime \prime }\left(\rho \right)\nabla \rho +\rho \nabla \Phi \right]. \end{array}$$

If we define the pressure P(ρ) such that

(A2) $$\begin{array}{c} P^{\prime }\left(\rho \right)=\frac{k_{B}T}{m}\rho C^{\prime \prime }\left(\rho \right), \end{array}$$

we get

(A3) $$\begin{array}{c} \xi \frac{\partial \rho }{\partial t}=\nabla \cdot \left(\nabla P+\rho \nabla \Phi \right). \end{array}$$

This generalized Smoluchowski equation, involving a generalized pressure P(ρ) instead of the ideal pressure $P_{\mathrm{id}}=\rho k_{B}T/m$, was introduced in [97]. The square of the velocity of sound is $c_{s}^{2}=P^{\prime }\left(\rho \right)$ and it reduces to $k_{B}T/m$ in the ideal case. Comparing Equation (A3) with Equation (39), we see that P(ρ)=D(ρ)ρ/χ. At equilibrium, we obtain the equation

(A4) $$\begin{array}{c} \nabla P+\rho \nabla \Phi =0, \end{array}$$

which can be interpreted as the condition of hydrostatic equilibrium stating that the pressure gradient equilibrates the mean field force. This equation can also be written as

(A5) $$\begin{array}{c} \frac{P^{\prime }\left(\rho \right)}{\rho }=-\frac{1}{\rho^{\prime }\left(\Phi \right)}=-\Phi^{\prime }\left(\rho \right), \end{array}$$

which is equivalent to Equation (30) if we make use of Equation (A2). We have

(A6) $$\begin{array}{c} \int \frac{P^{\prime }\left(\rho \right)}{\rho }\;d\rho =-\Phi ,\;P\left(\rho \right)=\int \Phi \left(\rho \right)\;d\rho -\rho \Phi \left(\rho \right). \end{array}$$

It is convenient to introduce the density of free energy

(A7) $$\begin{array}{c} f\left(\rho \right)=\frac{k_{B}T}{m}C\left(\rho \right), \end{array}$$

so that the free energy functional from Equation (14) takes the form

(A8) $$\begin{array}{c} F\left[\rho \right]=\int f\left(\rho \right)\;dr+\frac{1}{2}\int \rho \Phi \;dr. \end{array}$$

Equation (A2) then yields

(A9) $$\begin{array}{c} P^{\prime }\left(\rho \right)=\rho f^{\prime \prime }\left(\rho \right). \end{array}$$

If we define the enthalpy h(ρ) by dh=dP/ρ, we get $h\left(\rho \right)=f^{\prime }\left(\rho \right)=\int \left[P^{\prime }\left(\rho \right)/\rho \right]\;d\rho$. The equation of state P(ρ) is determined by the density of free energy from Equation (A7) according to

(A10) $$\begin{array}{c} P\left(\rho \right)=\rho^{2}{\left[\frac{f(\rho )}{\rho }\right]}^{\prime }=f^{\prime }\left(\rho \right)\rho -f\left(\rho \right). \end{array}$$

This relation, which can be rewritten as P=−d/d(1/ρ)(f/ρ), is the local version of the general thermodynamic relation P=−∂F/∂V defining the pressure. Inversely, the density of free energy is determined by the equation of state P(ρ) according to

(A11) $$\begin{array}{c} f\left(\rho \right)=\rho \int^{\rho }\frac{P(\rho^{\prime })}{{\rho^{\prime }}^{2}}\;d\rho^{\prime }=\rho \int^{\rho }\frac{P^{\prime }\left(\rho^{\prime }\right)}{\rho^{\prime }}\;d\rho^{\prime }-P\left(\rho \right). \end{array}$$

Using Equation (A11), the free energy from Equation (14) can be written as

(A12) $$\begin{array}{c} F\left[\rho \right]=\int \rho \int^{\rho }\frac{P(\rho^{\prime })}{{\rho^{\prime }}^{2}}\;d\rho^{\prime }dr+\frac{1}{2}\int \rho \Phi \;dr. \end{array}$$

According to Equations (A7) and (A10) we note that the pressure can be written as

(A13) $$\begin{array}{c} P\left(\rho \right)=\sigma \left(\rho \right)\frac{k_{B}T}{m}, \end{array}$$

where $\sigma \left(\rho \right)=C^{\prime }\left(\rho \right)\rho -C\left(\rho \right)$ is a function that depends only on the density.

### Appendix A.2. Weakly Inhomogeneous Systems

If the density varies weakly with the position, we find from Equation (6) that

(A14) $$\begin{array}{c} \Phi (r,t)≃-a\rho (r,t), \end{array}$$

where

(A15) $$\begin{array}{c} a=-\int u(r)\;dr. \end{array}$$

(If we go to the next order in the expansion, yielding Φ≃−aρ−KΔρ, we obtain a generalized Cahn-Hilliard equation associated with a square gradient free energy functional – see [50] for details and discussion.) In that case, the free energy functional from Equation (14) becomes

(A16) $$\begin{array}{c} F\left[\rho \right]=\frac{k_{B}T}{m}\int C\left(\rho \right)\;dr-\frac{1}{2}a\int \rho^{2}\;dr. \end{array}$$

The total density of free energy is

(A17) $$\begin{array}{c} f_{\mathrm{tot}}\left(\rho \right)=\frac{k_{B}T}{m}C\left(\rho \right)-\frac{1}{2}a\rho^{2}. \end{array}$$

If we introduce an external potential $\Phi_{\mathrm{ext}}$, the free energy functional is given by

(A18) $$\begin{array}{c} F\left[\rho \right]=\int f_{\mathrm{tot}}\left(\rho \right)dr+\frac{1}{2}\int \rho \Phi_{\mathrm{ext}}\;dr. \end{array}$$

On the other hand, the generalized Smoluchowski Equation (A1) takes the form

(A19) $$\begin{array}{c} \xi \frac{\partial \rho }{\partial t}=\nabla \cdot \left(\nabla P-a\rho \nabla \rho +\rho \nabla \Phi_{\mathrm{ext}}\right). \end{array}$$

It can be rewritten as

(A20) $$\begin{array}{c} \xi \frac{\partial \rho }{\partial t}=\nabla \cdot \left(\nabla P_{\mathrm{tot}}+\rho \nabla \Phi_{\mathrm{ext}}\right), \end{array}$$

where

(A21) $$\begin{array}{c} P_{\mathrm{tot}}\equiv P-\frac{1}{2}a\rho^{2}=\sigma \left(\rho \right)\frac{k_{B}T}{m}-\frac{1}{2}a\rho^{2} \end{array}$$

is the total pressure. It takes into account the contribution of the pressure *P* arising from short-range interactions (through the generalized entropy C(ρ)) and the contribution of the pressure $-\frac{1}{2}a\rho^{2}$ arising from long-range interactions [through the potential of interaction $u(|r-r^{\prime }|)$]. In particular, when

(A22) $$\begin{array}{c} P=\frac{\rho }{1-\rho /\rho_{*}}\frac{k_{B}T}{m}, \end{array}$$

corresponding to a generalized entropy of the form [110,111]

(A23) $$\begin{array}{c} C\left(\rho \right)=\rho \ln\left(\frac{\rho }{\rho_{*}-\rho }\right) \end{array}$$

taking excluded volume effects into account (this entropy can be obtained from a combinatorial analysis [110,111]), we recover the celebrated van der Waals [153] equation of state

(A24) $$\begin{array}{c} P_{\mathrm{tot}}=\frac{\rho }{1-\rho /\rho_{*}}\frac{k_{B}T}{m}-\frac{1}{2}a\rho^{2}. \end{array}$$

We note that Equation (A22) is exact for hard rods in one dimension [154], while it is only approximate for hard spheres in 3D. Other examples of generalized entropies and their corresponding equations of state (or the converse) are given in the following section and in [50,106].

### Appendix A.3. Examples of Generalized Entropies

For a given generalized entropy C(ρ), the equation of state P(ρ) can be obtained from Equation (A10) and the density–potential relation ρ(Φ) can be obtained from Equation (27). Inversely, for a given equation of state P(ρ), the generalized entropy C(ρ) can be obtained from Equation (A11) and the density–potential relation ρ(Φ) can be obtained from Equation (A6). For a given density–potential relation ρ(Φ), the generalized entropy can be obtained from Equation (29) and the equation of state can be obtained from Equation (A6).

(i) For the Boltzmann entropy

(A25) $$\begin{array}{c} C\left(\rho \right)=\rho \ln\left(\frac{\rho }{\rho_{*}}\right), \end{array}$$

we get an isothermal equation of state and an exponential density–potential relation

(A26) $$\begin{array}{c} P=\rho \frac{k_{B}T}{m},\;\rho =\rho_{*}e^{-\beta m\Phi +\alpha -1}. \end{array}$$

The Boltzmann entropy can be obtained from a standard combinatorial analysis.

(ii) For the Tsallis entropy [80]

(A27) $$\begin{array}{c} C\left(\rho \right)=\frac{1}{\gamma -1}\left[\rho_{*}{\left(\frac{\rho }{\rho_{*}}\right)}^{\gamma }-\rho \right], \end{array}$$

we get a polytropic equation of state and a power-law density–potential relation

(A28) $$\begin{array}{c} P=\rho_{*}{\left(\frac{\rho }{\rho_{*}}\right)}^{\gamma }\frac{k_{B}T}{m},\;\rho =\rho_{*}{\left(\frac{1}{\gamma }\right)}^{1/(\gamma -1)}{\left[(\gamma -1)(-\beta m\Phi +\alpha )+1\right]}_{+}^{1/(\gamma -1)}. \end{array}$$

The Boltzmann entropy from Equation (A25) is recovered for γ→1.

(iii) For the entropy [106]

(A29) $$\begin{array}{c} C\left(\rho \right)=\rho \ln\left(\rho /\rho_{*}\right)+\frac{\rho_{*}}{K}\left(1-K\rho /\rho_{*}\right)\ln\left(1-K\rho /\rho_{*}\right), \end{array}$$

we get

(A30) $$\begin{array}{c} P=-\frac{\rho_{*}}{K}\ln\left(1-K\rho /\rho_{*}\right)\frac{k_{B}T}{m},\;\rho =\frac{\rho_{*}}{e^{\beta m\Phi -\alpha }+K}. \end{array}$$

This entropy includes the Fermi–Dirac entropy (K=+1) [81,82,102,132] and the Bose–Einstein entropy (K=−1) [82,84]. These entropies can be obtained from a combinatorial analysis taking into account exclusion or inclusion constraints in position space. For $\rho \ll \rho_{*}$ we recover the Boltzmann entropy from Equation (A25).

(iv) For the entropy

(A31) $$\begin{array}{c} C\left(\rho \right)=\rho \ln\left(\rho /\rho_{*}\right)-\frac{\rho_{*}}{K}\left(1-K\rho /\rho_{*}\right)\ln\left(1-K\rho /\rho_{*}\right), \end{array}$$

we get

(A32) $$\begin{array}{c} P=\left[2\rho +\frac{\rho_{*}}{K}\ln\left(1-K\rho /\rho_{*}\right)\right]\frac{k_{B}T}{m},\;\rho =\frac{\rho_{*}}{2K}\left(1\pm \sqrt{1-4Ke^{-\beta m\Phi +\alpha -2}}\right). \end{array}$$

For $\rho \ll \rho_{*}$ we recover the Boltzmann entropy from Equation (A25).

(v) For the entropy of the logotropic gas [129]

(A33) $$\begin{array}{c} C\left(\rho \right)=A\rho_{*}\ln\left(\frac{\rho }{\rho_{*}}\right), \end{array}$$

we get

(A34) $$\begin{array}{c} P\left(\rho \right)=A\left[1-\ln\left(\frac{\rho }{\rho_{*}}\right)\right]\rho_{*}\frac{k_{B}T}{m},\;\rho =\frac{A\rho_{*}}{-\beta m\Phi +\alpha }. \end{array}$$

The logotropic equation of state, Equation (A34), has been used in a cosmological model [155].

(vi) For the entropy

(A35) $$\begin{array}{c} C\left(\rho \right)=-A\rho_{*}{\left[\ln\left(\frac{\rho_{*}}{\rho }\right)\right]}^{\delta }, \end{array}$$

we get

(A36) $$\begin{array}{c} P\left(\rho \right)=A{\left[\ln\left(\frac{\rho_{*}}{\rho }\right)\right]}^{\delta -1}\left[\delta +\ln\left(\frac{\rho_{*}}{\rho }\right)\right]\rho_{*}\frac{k_{B}T}{m}, \end{array}$$

(A37) $$\begin{array}{c} \rho =\rho_{*}e^{-\left(\delta -1\right)W\left[\frac{1}{\delta -1}{\left(\frac{-\beta m\Phi +\alpha }{\delta A}\right)}^{\frac{1}{\delta -1}}\right]}, \end{array}$$

where W(z) is the Lambert function defined by $We^{W}=z$. For δ=1, we recover the logotropic gas (see Equation (A33)).

(vii) For the entropy

(A38) $$\begin{array}{c} C\left(\rho \right)=A\rho_{*}{\left(\frac{\rho }{\rho_{*}}\right)}^{\gamma }\ln\left(\frac{\rho }{\rho_{*}}\right), \end{array}$$

we get

(A39) $$\begin{array}{c} P\left(\rho \right)=A{\left(\frac{\rho }{\rho_{*}}\right)}^{\gamma }\left[1+\left(\gamma -1\right)\ln\left(\frac{\rho }{\rho_{*}}\right)\right]\rho_{*}\frac{k_{B}T}{m}, \end{array}$$

(A40) $$\begin{array}{c} \rho =\rho_{*}\frac{1}{e^{1/\gamma }}e^{\frac{1}{\gamma -1}W\left[\frac{\gamma -1}{\gamma A}e^{\frac{\gamma -1}{\gamma }}\left(-\beta m\Phi +\alpha \right)\right]}. \end{array}$$

For γ=1 we recover the Boltzmann gas and for γ=0 we recover the logotropic gas. The entropy from Equation (A38) yields the Anton-Schmidt equation of state (see Equation (A50)).

(viii) For the entropy

(A41) $$\begin{array}{c} C\left(\rho \right)=-A\rho {\left[\ln\left(\frac{\rho_{*}}{\rho }\right)\right]}^{\delta }, \end{array}$$

we get

(A42) $$\begin{array}{c} P\left(\rho \right)=A\delta {\left[\ln\left(\frac{\rho_{*}}{\rho }\right)\right]}^{\delta -1}\rho \frac{k_{B}T}{m},\;\rho =\rho_{*}e^{-1-\sqrt{1-(\alpha -\beta m\Phi )/A}}\;\left(\delta =2\right). \end{array}$$

For δ=1 we recover the Boltzmann gas. The entropy from Equation (A41) has been applied to black hole physics [156].

(ix) For the entropy

(A43) $$\begin{array}{c} C\left(\rho \right)=-A\rho_{*}{\left(\frac{\rho }{\rho_{*}}\right)}^{\gamma }{\left[\ln\left(\frac{\rho_{*}}{\rho }\right)\right]}^{\delta }, \end{array}$$

we get

(A44) $$\begin{array}{c} P\left(\rho \right)=A{\left(\frac{\rho }{\rho_{*}}\right)}^{\gamma }{\left[\ln\left(\frac{\rho_{*}}{\rho }\right)\right]}^{\delta -1}\left[\delta -\left(\gamma -1\right)\ln\left(\frac{\rho_{*}}{\rho }\right)\right]\rho_{*}\frac{k_{B}T}{m}. \end{array}$$

This entropy contains all the particular cases described previously.

(x) For the Carnahan-Starling equation of state [157]

(A45) $$\begin{array}{c} P=\rho \frac{1+\eta +\eta^{2}-\eta^{3}}{{\left(1-\eta \right)}^{3}}\frac{k_{B}T}{m}, \end{array}$$

where $\eta =\rho /\rho_{*}$, we get

(A46) $$\begin{array}{c} C\left(\rho \right)=\rho \left[\ln\left(\rho /\rho_{*}\right)+\frac{3-2\eta }{{\left(1-\eta \right)}^{2}}\right]. \end{array}$$

The Carnahan-Starling equation of state provides a very good approximation of the equation of state of a gas of hard spheres in 3D. For $\rho \ll \rho_{*}$ we recover the isothermal equation of state $P=\rho k_{B}T/m$.

(xi) For the equation of state

(A47) $$\begin{array}{c} P={\left(\sqrt{1+\rho /\rho_{*}}-1\right)}^{2}\rho_{*}\frac{k_{B}T}{m}, \end{array}$$

we get

(A48) $$\begin{array}{c} C\left(\rho \right)=-\rho +2\rho_{*}\sqrt{1+\rho /\rho_{*}}+2\rho \ln\left(1+\sqrt{1+\rho /\rho_{*}}\right), \end{array}$$

(A49) $$\begin{array}{c} \rho =\rho_{*}\left[e^{-\beta m\Phi +\alpha }-2e^{\frac{1}{2}\left(-\beta m\Phi +\alpha \right)}\right]. \end{array}$$

This equation of state has been used in a model of supernovae [158]. For $\rho \gg \rho_{*}$ we obtain the isothermal equation of state $P=\rho k_{B}T/m$ (Boltzmann) and for $\rho \ll \rho_{*}$ we obtain the polytropic equation of state $P=\frac{1}{4}\frac{\rho^{2}}{\rho_{*}}\frac{k_{B}T}{m}$ of index γ=2 (Tsallis).

(xii) For the Anton-Schmidt equation of state [159]

(A50) $$\begin{array}{c} P\left(\rho \right)=A{\left(\frac{\rho }{\rho_{*}}\right)}^{\gamma }\ln\left(\frac{\rho }{\rho_{*}}\right)\rho_{*}\frac{k_{B}T}{m}, \end{array}$$

we get

(A51) $$\begin{array}{c} C\left(\rho \right)=\frac{A}{{\left(\gamma -1\right)}^{2}}{\left(\frac{\rho }{\rho_{*}}\right)}^{\gamma }\left[\left(\gamma -1\right)\ln\left(\frac{\rho }{\rho_{*}}\right)-1\right]. \end{array}$$

This corresponds to the entropy from Equation (A38). For γ=0 we recover the logotropic gas.

(xiii) For the equation of state

(A52) $$\begin{array}{c} P\left(\rho \right)=-A{\left[\ln\left(\frac{\rho_{*}}{\rho }\right)\right]}^{\delta }\rho \frac{k_{B}T}{m}, \end{array}$$

we get

(A53) $$\begin{array}{c} C\left(\rho \right)=\frac{A\rho }{\delta +1}\;{\left[\ln\left(\frac{\rho_{*}}{\rho }\right)\right]}^{\delta +1}. \end{array}$$

This corresponds to the entropy from Equation (A41). For δ=0 we recover the Boltzmann gas.

(xiv) For the equation of state

(A54) $$\begin{array}{c} P\left(\rho \right)=-A{\left(\frac{\rho }{\rho_{*}}\right)}^{\gamma }{\left[\ln\left(\frac{\rho_{*}}{\rho }\right)\right]}^{\delta }\rho_{*}\frac{k_{B}T}{m}, \end{array}$$

we get

(A55) $$\begin{array}{c} C\left(\rho \right)=-\frac{A\rho }{{\left(\gamma -1\right)}^{\delta +1}}\;\Gamma \left[1+\delta ,\left(\gamma -1\right)\ln\left(\frac{\rho_{*}}{\rho }\right)\right], \end{array}$$

where $\Gamma \left(a,x\right)=\int_{x}^{+\infty }e^{-t}t^{a-1}\;dt$ is the incomplete Gamma function. This equation of state contains all the particular cases described previously.

(xv) For a stretched exponential density–potential relation

(A56) $$\begin{array}{c} \rho =\rho_{*}\;e^{-{\left(\beta m\Phi -\alpha \right)}^{\delta }}, \end{array}$$

we get

(A57) $$\begin{array}{c} C\left(\rho \right)=-\rho_{*}\Gamma \left[\frac{\delta +1}{\delta },\ln\left(\frac{\rho_{*}}{\rho }\right)\right],\;P=\frac{1}{\delta }\;\Gamma \left[\frac{1}{\delta },\ln\left(\frac{\rho_{*}}{\rho }\right)\right]\rho_{*}\frac{k_{B}T}{m}. \end{array}$$

## Appendix B. The Functional Fokker-Planck Equation

### Appendix B.1. Derivation of the Functional Fokker-Planck Equation

The generalized stochastic Smoluchowski Equation (83) can be written as

(A58) $$\begin{array}{c} \frac{\partial \rho }{\partial t}=\nabla \cdot \left[\chi g\left(\rho \right)\nabla \frac{\delta F}{\delta \rho }\right]+\zeta \left(r,t\right), \end{array}$$

where

(A59) $$\begin{array}{c} \zeta \left(r,t\right)=\nabla \cdot \left[\sqrt{2\chi k_{B}Tg\left(\rho \right)}\;R\left(r,t\right)\right] \end{array}$$

is a Gaussian white noise with zero mean and correlation function

(A60) $$\begin{array}{c} \langle \zeta \left(r,t\right)\zeta \left(r^{\prime },t^{\prime }\right)\rangle =2\chi k_{B}T\left(\nabla \cdot \nabla^{\prime }\right)g\left(\rho \right)\delta \left(r-r^{\prime }\right)\delta \left(t-t^{\prime }\right). \end{array}$$

Equation (A58) can be interpreted as a Langevin equation for ρ(r,t). We now turn to the derivation of the Fokker-Planck equation for the density distribution functional P[ρ,t]. Since the noise in Equation (A58) is multiplicative (it depends on ρ), we must specify the meaning of the stochastic integral associated with the random noise ζ(r,t). From the requirement that the Fokker-Planck equation should have the stationary distribution function $P_{\mathrm{eq}}\propto \exp\left(-\beta F\left[\rho \right]\right)$ the integral will be treated as an Ito type one. If we express the increment of density ρ(r) between *t* and t+Δt by Δρ(r) we see from Equations (A58) and (A60) that

(A61) $$\begin{array}{c} \frac{\langle \Delta \rho (r)\rangle }{\Delta t}=-\nabla \cdot J=\nabla \cdot \left[\chi g\left(\rho \right)\nabla \frac{\delta F}{\delta \rho }\right] \end{array}$$

and

(A62) $$\begin{array}{c} \frac{\langle \Delta \rho \left(r\right)\Delta \rho \left(r^{\prime }\right)\rangle }{2\Delta t}=\chi k_{B}T\left(\nabla \cdot \nabla^{\prime }\right)g\left(\rho \right)\delta \left(r-r^{\prime }\right). \end{array}$$

Denoting bt P[ρ(r),t] the probability functional for the density field ρ(r) at time *t*, the Fokker-Planck equation associated with the Langevin Equation (A58) is given by

(A63) $$\begin{array}{c} \begin{array}{c} \frac{\partial P}{\partial t}\left[\rho ,t\right]=-\int dr\;\frac{\delta }{\delta \rho (r)}\left\{P\nabla \cdot \left[\chi g\left(\rho \right)\nabla \frac{\delta F}{\delta \rho }\right]\right\}+\int drdr^{\prime }\;\frac{\delta^{2}}{\delta \rho \left(r\right)\delta \rho \left(r^{\prime }\right)}\left[P\chi k_{B}T\left(\nabla \cdot \nabla^{\prime }\right)g\left(\rho \right)\delta \left(r-r^{\prime }\right)\right]. \end{array} \end{array}$$

The second term can be rewritten as

(A64) $$\begin{array}{c} \begin{array}{cc} & \int dr\;\frac{\delta }{\delta \rho (r)}\nabla \cdot \int dr^{\prime }\;\frac{\delta }{\delta \rho (r^{\prime })}\left[P\chi k_{B}T\nabla^{\prime }g\left(\rho \right)\delta \left(r-r^{\prime }\right)\right]\\ = & -\int dr\;\frac{\delta }{\delta \rho (r)}\nabla \cdot \int dr^{\prime }\;\nabla^{\prime }\left(\frac{\delta P}{\delta \rho (r^{\prime })}\right)\chi k_{B}Tg\left(\rho \right)\delta \left(r-r^{\prime }\right)\\ = & -\int dr\;\frac{\delta }{\delta \rho (r)}\nabla \cdot \chi k_{B}Tg\left(\rho \right)\nabla \frac{\delta P}{\delta \rho (r)}. \end{array} \end{array}$$

This yields the following functional Fokker-Planck equation

(A65) $$\begin{array}{c} \begin{array}{ccc} \frac{\partial P}{\partial t}\left[\rho ,t\right] & = & -\int dr\;\frac{\delta }{\delta \rho (r)}\left\{\nabla \cdot \left[\chi g\left(\rho \right)\nabla \frac{\delta F}{\delta \rho (r)}\right]P+k_{B}T\nabla \cdot \chi g\left(\rho \right)\nabla \frac{\delta P}{\delta \rho (r)}\right\}\\ & = & -\int dr\;\frac{\delta }{\delta \rho (r)}\left\{\nabla \cdot \chi g\left(\rho \right)\nabla \left[k_{B}T\frac{\delta }{\delta \rho (r)}+\frac{\delta F}{\delta \rho (r)}\right]P\right\}, \end{array} \end{array}$$

which is Equation (84). It can be written as

(A66) $$\begin{array}{c} \frac{\partial P}{\partial t}\left[\rho ,t\right]=-\int dr\;\frac{\delta }{\delta \rho (r)}\nabla \cdot J\left[P\right], \end{array}$$

where

(A67) $$\begin{array}{c} J\left[P\right]=\chi g\left(\rho \right)\nabla \left(k_{B}T\frac{\delta }{\delta \rho }+\frac{\delta F}{\delta \rho }\right)P \end{array}$$

is the functional Fokker-Planck current. The equilibrium solution of Equation (84) is given by Equation (17) as can be checked by direct substitution. Therefore, the Langevin Equation (83) samples, at equilibrium, the density field ρ(r) according to the weight exp(−βF[ρ]).

### Appendix B.2. H-Theorem

We introduce the free energy functional

(A68) $$\begin{array}{c} F\left[P\right]=\int P\left[\rho ,t\right]F\left[\rho \right]\;D\rho +k_{B}T\int P\left[\rho ,t\right]\ln P\left[\rho ,t\right]\;D\rho . \end{array}$$

Using the Fokker-Planck Equation (A65), the rate of dissipation of free energy is

(A69) $$\begin{array}{c} \begin{array}{ccc} \frac{d}{dt}F\left[P\right] & = & \int D\rho \;\left(k_{B}T\ln P+k_{B}T+F\right)\frac{\partial P}{\partial t}\\ & = & -\int drD\rho \;\left(k_{B}T\ln P+k_{B}T+F\right)\frac{\delta }{\delta \rho }\nabla \cdot J\left[P\right]\\ & = & \int drD\rho \;\left(\frac{k_{B}T}{P}\frac{\delta P}{\delta \rho }+\frac{\delta F}{\delta \rho }\right)\nabla \cdot J\left[P\right]\\ & = & -\int drD\rho \;\nabla \left(\frac{k_{B}T}{P}\frac{\delta P}{\delta \rho }+\frac{\delta F}{\delta \rho }\right)\cdot J\left[P\right]\\ & = & -\int drD\rho \;\frac{\chi g(\rho )}{P}{\left[\nabla \left(k_{B}T\frac{\delta }{\delta \rho }+\frac{\delta F}{\delta \rho }\right)P\right]}^{2}. \end{array} \end{array}$$

This yields the *H*-theorem:

(A70) $$\begin{array}{c} \dot{F}\leq 0,\;\dot{F}=0\;\Leftrightarrow \;J=0. \end{array}$$

Therefore F decreases in time monotonically until P[ρ,t] takes the form of Equation (17).

If we consider the average free energy

(A71) $$\begin{array}{c} \langle F\rangle =\int P[\rho ,t]F[\rho ]\;D\rho , \end{array}$$

we find that

(A72) $$\begin{array}{c} \begin{array}{ccc} \frac{d}{dt}\langle F\rangle & = & \int D\rho \;F\frac{\partial P}{\partial t}\\ & = & -\int drD\rho \;F\frac{\delta }{\delta \rho }\nabla \cdot J\left[P\right]\\ & = & \int drD\rho \;\frac{\delta F}{\delta \rho }\nabla \cdot J\left[P\right]\\ & = & -\int drD\rho \;\nabla \frac{\delta F}{\delta \rho }\cdot J\left[P\right]\\ & = & -\int drD\rho \;\chi g\left(\rho \right){\left(\nabla \frac{\delta F}{\delta \rho }\right)}^{2}P-k_{B}T\int drD\rho \;\chi g\left(\rho \right)\nabla \frac{\delta F}{\delta \rho }\cdot \nabla \frac{\delta P}{\delta \rho }. \end{array} \end{array}$$

The first term in the right hand side of the last line of Equation (A72) is the rate of dissipation of free energy from Equation (57) without fluctuations; it is always negative. The second term arises from the effect of fluctuations; it may be of any sign. Therefore, we do not have $\frac{d}{dt}\langle F\rangle \leq 0$ for the average free energy in the presence of fluctuations (noise). This is expected since the fluctuations can allow the free energy to increase. In this manner the system can cross the barrier of free energy played by the “critical nucleus” and trigger the transition from the metastable state to the stable state. This corresponds to the uphill path in the instanton theory (see Section 4.2). In conclusion, the fluctuations prevent the density field ρ(r,t) from being trapped in a local minimum of free energy F[ρ] and allow it to explore the complete free energy landscape.

### Appendix B.3. Onsager’s Linear Thermodynamics and Maximum Free Energy Dissipation Principle

The rate of dissipation of free energy is (see Equation (A69))

(A73) $$\begin{array}{c} \dot{F}\left[J\right]=-\int drD\rho \;\nabla \left(\frac{k_{B}T}{P}\frac{\delta P}{\delta \rho }+\frac{\delta F}{\delta \rho }\right)\cdot J\left[P\right]. \end{array}$$

Using arguments of linear thermodynamics similar to those of Section 3.8, the foregoing equation induces us to introduce a current of the form

(A74) $$\begin{array}{c} J\left[P\right]=\chi \left(\rho ,t\right)\nabla \left(k_{B}T\frac{\delta }{\delta \rho }+\frac{\delta F}{\delta \rho }\right)P, \end{array}$$

with χ(ρ,t)≥0, in order to get the *H*-theorem $\dot{F}\leq 0$. Equation (A74) coincides with the current from Equation (A67) with χ(ρ,t)=χg(ρ). Therefore, the functional Fokker-Planck Equation (A65) is consistent with Onsager’s linear thermodynamics. We can also obtain the Fokker-Planck current from Equation (A67) by a maximum free energy dissipation principle like in Section 3.9. To that purpose, we introduce the dissipation function

(A75) $$\begin{array}{c} E_{d}\left[J\right]=\int drD\rho \;\frac{J^{2}}{\chi g(\rho )P}. \end{array}$$

We can then check that the maximization problem

(A76) $$\begin{array}{c} \max_{J}\left\{-\dot{F}\left[J\right]-E_{d}\left[J\right]\right\} \end{array}$$

yields the Fokker-Planck current from Equation (A67).

## Appendix C. Density Functional Theory for Simple Liquids

In this Appendix, we review the main results obtained in the density functional theory of simple liquids and discuss the connection with our approach.

### Appendix C.1. The N-body Smoluchowski Equation

Let us consider a system of *N* Brownian particles in interaction in the strong friction limit ξ→+∞. In this Appendix, small-scale (microscopic) constraints are taken into account in the potential of interaction. The dynamics of the particles is described by the stochastic Langevin equations [23,62,77]

(A77) $$\begin{array}{c} \frac{dr_{i}}{dt}=-\frac{\chi }{m}\nabla_{i}U\left(r_{1},\ldots ,r_{N}\right)+\sqrt{2D}\;R_{i}\left(t\right), \end{array}$$

where $U(r_{1},\ldots ,r_{N})$ is the potential of interaction and $R_{i}\left(t\right)$ is a Gaussian white noise satisfying $\langle R_{i}\left(t\right)\rangle =0$ and $\langle R_{i}^{a}\left(t\right)R_{j}^{b}\left(t^{\prime }\right)\rangle =\delta_{ij}\delta_{ab}\delta \left(t-t^{\prime }\right)$. The evolution of the *N*-body distribution function $P_{N}\left(r_{1},\ldots ,r_{N},t\right)$ is governed by the *N*-body Smoluchowski equation [22,23,59,62,160]

(A78) $$\begin{array}{c} \frac{\partial P_{N}}{\partial t}=\sum_{i=1}^{N}\frac{\partial }{\partial r_{i}}\cdot \left[D\frac{\partial P_{N}}{\partial r_{i}}+\frac{\chi }{m}P_{N}\frac{\partial }{\partial r_{i}}U\left(r_{1},\ldots ,r_{N}\right)\right].\; \end{array}$$

Using the Einstein relation from Equation (2), we find that the equilibrium solution of the *N*-body Smoluchowski equation is the canonical distribution [62,161]

(A79) $$\begin{array}{c} P_{N}\left(r_{1},\ldots ,r_{N}\right)=\frac{1}{Z(\beta )}e^{-\beta U(r_{1},\ldots ,r_{N})}, \end{array}$$

where Z(β) is the partition function determined by the normalization condition $I\equiv \int P_{N}\;dr_{1}\ldots dr_{N}=1$. The *N*-body distribution from Equation (A79) corresponds to the statistical equilibrium state of the Brownian particles in the canonical ensemble. It gives the probability density of the microstate $\left\{r_{1},\ldots ,r_{N}\right\}$.

The proper thermodynamical potential in the canonical ensemble is the free energy

(A80) $$\begin{array}{c} F\left[P_{N}\right]=E\left[P_{N}\right]-TS\left[P_{N}\right], \end{array}$$

where the energy and the entropy of the *N*-body system are given by [23,161]

(A81) $$E\left[P_{N}\right]=\frac{3}{2}Nk_{B}T+\int P_{N}U\;dr_{1}\ldots dr_{N},$$

(A82) $$\begin{array}{c} S\left[P_{N}\right]=-k_{B}\int P_{N}\ln\left(\frac{h^{3N}}{m^{3N}}N!\;P_{N}\right)\;dr_{1}\ldots dr_{N}+\frac{3}{2}Nk_{B}\ln\left(\frac{2\pi k_{B}T}{m}\right)+\frac{3}{2}Nk_{B}. \end{array}$$

The free energy is explicitly given by

(A83) $$\begin{array}{c} F\left[P_{N}\right]=\int P_{N}U\;dr_{1}\ldots dr_{N}+k_{B}T\int P_{N}\ln\left(\frac{h^{3N}}{m^{3N}}N!\;P_{N}\right)\;dr_{1}\ldots dr_{N}-\frac{3}{2}Nk_{B}T\ln\left(\frac{2\pi k_{B}T}{m}\right).\; \end{array}$$

The *N*-body Smoluchowski equation satisfies an H-theorem for the free energy defined by Equation (A83). Indeed, a simple calculation shows that [23]

(A84) $$\dot{F}=-\sum_{i=1}^{N}\int \frac{m}{\xi P_{N}}{\left(\frac{k_{B}T}{m}\frac{\partial P_{N}}{\partial r_{i}}+\frac{1}{m}P_{N}\frac{\partial U}{\partial r_{i}}\right)}^{2}\;dr_{1}\ldots dr_{N}.$$

Therefore, $\dot{F}\leq 0$ and $\dot{F}=0$ if, and only if, the term in parenthesis vanishes. This leads to the canonical distribution from Equation (A79). Because of the H-theorem, the system converges towards the canonical distribution from Equation (A79) for t→+∞ (provided that Z<+∞). The canonical distribution from Equation (A79) is the (unique) minimum of $F[P_{N}]$ satisfying the normalization condition constraint. Indeed, the cancelation of the first variations δF−μδI=0, where μ is a Lagrange multiplier, returns Equation (A79) and the second variations $\delta^{2}F=\frac{1}{2}\int \frac{{\left(\delta P_{N}\right)}^{2}}{P_{N}}\;dr_{1}\ldots dr_{N}\geq 0$ are positive.

### Appendix C.2. The Deterministic Dynamical Density Functional Theory

The preceding results are completely general. From now on, we assume that the particles interact via a binary potential so that $U\left(r_{1},\ldots ,r_{N}\right)=\sum_{i<j}m^{2}u_{ij}$ where $u_{ij}=u(|r_{i}-r_{j}\left|\right)$. Starting from the *N*-body Smoluchowski Equation (A78) and writing down the equivalent of the Bogoliubov-Born-Green-Kirkwood-Yvon (BBGKY) hierarchy for the reduced probability distributions $P_{j}\left(r_{1},\ldots ,r_{j},t\right)$, we find that the equation for the average density (or one-body distribution function) of Brownian particles $\rho \left(r,t\right)=NmP_{1}\left(r,t\right)$ is [22,23,59,62,160]

(A85) $$\begin{array}{c} \frac{\partial \rho }{\partial t}=\nabla \cdot \left[D\nabla \rho +\chi \int \rho_{2}\left(r,r^{\prime },t\right)\nabla u(|r-r^{\prime }\left|\right)\;dr^{\prime }\right], \end{array}$$

where $\rho_{2}\left(r,r^{\prime },t\right)=N\left(N-1\right)m^{2}P_{2}\left(r,r^{\prime },t\right)$ is the two-body distribution function. At equilibrium, we get the Yvon–Born–Green (YBG) equation [162,163]

(A86) $$\begin{array}{c} \frac{k_{B}T}{m}\nabla \rho +\int \rho_{2}\left(r,r^{\prime }\right)\nabla u(|r-r^{\prime }\left|\right)\;dr^{\prime }=0. \end{array}$$

This equation can be obtained either as the equilibrium solution of Equation (A85) or by considering the first equation in the hierarchy for the reduced probability distributions $P_{j}\left(r_{1},\ldots ,r_{j}\right)$ obtained from the canonical distribution from Equation (A79) [23,62,161].

On the other hand, it can be shown [55] that the equilibrium density ρ(r) minimizes a certain free energy functional F[ρ] at fixed mass, i.e.,

(A87) $$\begin{array}{c} \min_{\rho (r)}\;\left\{F\left[\rho \right]\;|\;M\left[\rho \right]=M\right\}. \end{array}$$

This free energy functional can be written as

(A88) $$\begin{array}{c} F\left[\rho \right]=F_{\mathrm{id}}\left[\rho \right]+F_{\mathrm{ex}}\left[\rho \right], \end{array}$$

where

(A89) $$\begin{array}{c} F_{\mathrm{id}}=\frac{k_{B}T}{m}\int \rho \left[\ln\left(\lambda^{3}\frac{\rho }{m}\right)-1\right]\;dr \end{array}$$

is the ideal Bolzmann free energy (where $\lambda =h/\sqrt{2\pi mk_{B}T}$ is the de Broglie wavelength) and $F_{\mathrm{ex}}\left[\rho \right]$ is the excess free energy which takes the correlations into account. The equilibrium states are given by the condition

(A90) $$\begin{array}{c} \delta F-\frac{\mu }{m}\delta M=0\;\Leftrightarrow \;\frac{\delta F}{\delta \rho }=\frac{\mu }{m}, \end{array}$$

where μ is a Lagrange multiplier interpreted as a chemical potential. Using Equation (A89), Equation (A90) can be written as

(A91) $$\begin{array}{c} \frac{k_{B}T}{m}\ln\left(\lambda^{3}\frac{\rho }{m}\right)+\frac{\delta F_{\mathrm{ex}}}{\delta \rho }=\frac{\mu }{m}. \end{array}$$

Taking the gradient of this relation, we get [164,165]

(A92) $$\begin{array}{c} \frac{k_{B}T}{m}\nabla \rho +\rho \nabla \frac{\delta F_{\mathrm{ex}}}{\delta \rho }=0. \end{array}$$

Comparing Equation (A92) with Equation (A86), we find that [55]

(A93) $$\begin{array}{c} \int \rho_{2}\left(r,r^{\prime }\right)\nabla u(|r-r^{\prime }\left|\right)\;dr^{\prime }=\rho \left(r\right)\nabla \frac{\delta F_{\mathrm{ex}}}{\delta \rho }\left[\rho \left(r\right)\right]. \end{array}$$

This equation is exact at equilibrium. The idea of the dynamical density functional theory is to extend this relation out-of-equilibrium, i.e., to make the approximation [60,61]

(A94) $$\begin{array}{c} \int \rho_{2}\left(r,r^{\prime },t\right)\nabla u(|r-r^{\prime }\left|\right)\;dr^{\prime }=\rho \left(r,t\right)\nabla \cdot \frac{\delta F_{\mathrm{ex}}}{\delta \rho }\left[\rho \left(r,t\right)\right], \end{array}$$

where $F_{\mathrm{ex}}\left[\rho \right]$ is the equilibrium excess free energy. This closure is equivalent to assuming that the two-body dynamic correlations are the same as those in an equilibrium fluid with the same one-body density profile. Substituting this relation into Equation (A85), we obtain the gradient flow equation [60,61]

(A95) $$\begin{array}{c} \frac{\partial \rho }{\partial t}=\nabla \cdot \left(\chi \rho \nabla \frac{\delta F}{\delta \rho }\right). \end{array}$$

We note that the mobility is proportional to the density ρ(r,t).

Bibliographic note: The gradient flow Equation (A95) was obtained from the method described above by Marconi and Tarazona [60,61]. However, this equation was introduced earlier by several authors from (i) Onsager’s linear thermodynamics [51,54,55,57,58,59,74,75,76]; (ii) the strong friction limit of the Kramers equation [52,53]; (iii) the projection operator formalism [56]. This equation can be viewed as a generalization of the Cahn-Hilliard equation based on the square gradient functional [166,167].

### Appendix C.3. The Stochastic Dynamical Density Functional Theory

Starting from the Langevin Equation (A77), Dean [77] has shown that the discrete density $\rho_{d}\left(r,t\right)=\sum_{i}m\delta \left(r-r_{i}\left(t\right)\right)$ of the Brownian particles, which is a sum of Dirac distributions, is governed by a stochastic equation of the form

(A96) $$\begin{array}{c} \frac{\partial \rho_{d}}{\partial t}=\nabla \cdot \left(\chi \rho_{d}\nabla \frac{\delta F_{d}}{\delta \rho_{d}}\right)+\nabla \cdot \left[\sqrt{2\chi k_{B}T\rho_{d}}\;R\left(r,t\right)\right], \end{array}$$

where R(r,t) is a Gaussian white noise satisfying 〈R(r,t)〉=0 and $\langle R_{a}\left(r,t\right)R_{b}\left(r^{\prime },t^{\prime }\right)\rangle =\delta_{ab}\delta \left(r-r^{\prime }\right)\delta \left(t-t^{\prime }\right)$, and $F_{d}\left[\rho_{d}\right]$ is the discrete free energy

(A97) $$\begin{array}{c} F_{d}=\frac{k_{B}T}{m}\int \rho_{d}\ln\rho_{d}\;dr+\frac{1}{2}\int \rho_{d}\left(r,t\right)u(|r-r^{\prime }\left|\right)\rho_{d}\left(r^{\prime },t\right)\;drdr^{\prime }. \end{array}$$

The Dean equation can be written explicitly as

(A98) $$\begin{array}{c} \frac{\partial \rho_{d}}{\partial t}=\nabla \cdot \left[D\nabla \rho_{d}+\chi \rho_{d}\nabla \int \rho_{d}\left(r^{\prime },t\right)u(|r-r^{\prime }\left|\right)\;dr^{\prime }\right]+\nabla \cdot \left[\sqrt{2\chi k_{B}T\rho_{d}}\;R\left(r,t\right)\right]. \end{array}$$

This equation is exact in the sense that it is equivalent to the Langevin Equation (A77) or to the *N*-body Smoluchowski Equation (A78). If we take the ensemble average of the Dean Equation (A98), we obtain [60,61]

(A99) $$\begin{array}{c} \frac{\partial \rho }{\partial t}=\nabla \cdot \left[D\nabla \rho +\chi \int \langle \rho_{d}\left(r,t\right)\rho_{d}\left(r^{\prime },t\right)\rangle \nabla u(|r-r^{\prime }\left|\right)\;dr^{\prime }\right], \end{array}$$

which coincides with Equation (A85) after noting that $\langle \rho_{d}\left(r,t\right)\rho_{d}\left(r^{\prime },t\right)\rangle =\rho \left(r,t\right)m\delta \left(r-r^{\prime }\right)+\rho_{2}\left(r,r^{\prime },t\right)$. Then, using the Ansatz from Equation (A94), we obtain the deterministic Smoluchowski Equation (A95). Alternatively, if we use a mesoscopic approach and coarse-grain the Dean Equation (A96), while keeping track of fluctuations, we obtain the stochastic Smoluchowski equation [78]

(A100) $$\begin{array}{c} \frac{\partial \rho }{\partial t}=\nabla \cdot \left(\chi \rho \nabla \frac{\delta F}{\delta \rho }\right)+\nabla \cdot \left[\sqrt{2\chi k_{B}T\rho }\;R\left(r,t\right)\right], \end{array}$$

where, now, *F* is the free energy from Equation (A88) which is obviously very different from Equation (A97). This equation can also be obtained from the theory of fluctuating hydrodynamics [14,63]. While they have a similar mathematical structure, the Dean Equation (A96) and the stochastic Smoluchowski Equation (A100) are physically very different from each other as explained in Section 4.1 (see also the discussion in [78]).

Bibliographic note: The mesoscopic Smoluchowski Equation (A100) was first obtained by Munakata [74,75,76] by taking the overdamped limit of the hydrodynamic equations derived by Kirkpatrick and Wolynes [58]. This equation can be viewed as a generalization of the stochastic Cahn-Hilliard equation based on the square gradient functional [168,169]. The functional Fokker-Planck equation associated with the Dean Equation (A96) can be written as Equation (84) with g(ρ)=ρ and with the free energy from Equation (A97). It was first written by Frusawa and Hayakawa [170]. The functional Fokker-Planck equation associated with the mesoscopic Smoluchowski Equation (A100) can be written as Equation (84) with g(ρ)=ρ and with the free energy from Equation (A88). It was first written by Munakata [74,75,76] and Kawasaki [171]. This equation can be viewed as a generalization of the functional Fokker-Planck equation associated with the stochastic Cahn-Hilliard equation based on the square gradient functional [172].

### Appendix C.4. The Direct Correlation Function

The preceding equations are general and, to some extent, rigorous (they rely on the approximation from Equation (A95) which has been shown to be very good in many cases). The difficulty now is to obtain the expression of the excess free energy functional $F_{\mathrm{ex}}\left[\rho \right]$. This functional is known exactly only for some simple systems such as hard rods in one dimension [173], but approximate expressions can be obtained in more general situations (see [152] for some standard methods).

In many models of liquids, the excess free energy is written under the quadratic form

(A101) $$\begin{array}{c} F_{\mathrm{ex}}\left[\rho \right]=\frac{1}{2}\int \rho \left(r,t\right)u_{\mathrm{eff}}(|r-r^{\prime }\left|\right)\rho \left(r^{\prime },t\right)\;drdr^{\prime }, \end{array}$$

where $u_{\mathrm{eff}}(|r-r^{\prime }\left|\right)$ is an effective potential of interaction that takes correlations into account. It usually differs from the bare potential of interaction $u(|r-r^{\prime }|)$. It is related to the direct correlation function

(A102) $$\begin{array}{c} c\left(r,r^{\prime }\right)=-\beta m^{2}\frac{\delta^{2}F_{\mathrm{ex}}}{\delta \rho \left(r\right)\delta \rho \left(r^{\prime }\right)} \end{array}$$

by

(A103) $$\begin{array}{c} u_{\mathrm{eff}}(|r-r^{\prime }\left|\right)=-\frac{k_{B}T}{m^{2}}c(|r-r^{\prime }\left|\right). \end{array}$$

This relation first appeared in a work of Zwanzig [174]. In general, the direct correlation function $c(r,r^{\prime })$ can be obtained either experimentally or by approximate methods. It then determines the effective potential of interaction $u_{\mathrm{eff}}(|r-r^{\prime }\left|\right)$ through Equation (A103) and the excess free energy through Equation (A101). The total free energy can be written as

(A104) $$\begin{array}{c} F\left[\rho \right]=\frac{k_{B}T}{m}\int \rho \left[\ln\left(\lambda^{3}\frac{\rho }{m}\right)-1\right]\;dr+\frac{1}{2}\int \rho \left(r,t\right)u_{\mathrm{eff}}(|r-r^{\prime }\left|\right)\rho \left(r^{\prime },t\right)\;drdr^{\prime }. \end{array}$$

We see that the results obtained for systems with long-range interactions in the mean field approximation (as described in this paper) can be applied to simple liquids provided that the potential of interaction $u(|r-r^{\prime }|)$ is replaced by the effective potential of interaction $u_{\mathrm{eff}}(|r-r^{\prime }\left|\right)$. In that case, we must also take $C\left(\rho \right)=\rho \ln\left(\lambda^{3}\frac{\rho }{m}\right)$ (ideal Boltzmann entropy) and g(ρ)=ρ (constant mobility). For systems with purely long-range interactions, we have [64]

(A105) $$\begin{array}{c} u_{\mathrm{eff}}(|r-r^{\prime }\left|\right)=u_{\mathrm{LR}}(|r-r^{\prime }\left|\right). \end{array}$$

If we now consider systems with short-range interactions and long-range interactions, we can apply the results of this paper by replacing *u* by [62,63]

(A106) $$\begin{array}{c} u_{\mathrm{tot}}(|r-r^{\prime }\left|\right)=-\frac{k_{B}T}{m^{2}}c(|r-r^{\prime }\left|\right)+u_{\mathrm{LR}}(|r-r^{\prime }\left|\right). \end{array}$$

The first term is the effective potential of interaction $u_{\mathrm{eff}}(|r-r^{\prime }\left|\right)$ taking into account correlations due to short-range interactions as described in this Appendix and the second term takes into account long-range interactions in a mean field approximation. To determine the direct correlation function $c(|r-r^{\prime }|)$ one can use, for example, the Percus-Yevick [175] integral equation. Its solution is known exactly for the special case of a fluid of hard spheres [176,177,178] but approximate expressions can be obtained in more general situations.

### Appendix C.5. Weakly Inhomogeneous Systems

In this Appendix, we first assume that the potential of interaction is short-ranged. If the density varies weakly with the position we can write the excess free energy as

(A107) $$\begin{array}{c} F_{\mathrm{ex}}\left[\rho \right]=\int f_{\mathrm{ex}}\left(\rho \right)\;dr. \end{array}$$

The excess free energy density $f_{\mathrm{ex}}\left(\rho \right)$ can be obtained from the model discussed in Appendix C.4 as follows. First, we have to realize that, in general, the direct correlation function $c(|r-r^{\prime }|;\rho )$ depends explicitly on the density, even though we ignore this dependence when we take functional derivatives. The excess free energy can then be written as

(A108) $$\begin{array}{c} F_{\mathrm{ex}}\left[\rho \right]=\frac{1}{2}\int \rho \left(r,t\right)u_{\mathrm{eff}}(|r-r^{\prime }\left|;\rho \right)\rho \left(r^{\prime },t\right)\;drdr^{\prime }. \end{array}$$

If the density varies weakly with the position, we get

(A109) $$\begin{array}{c} F_{\mathrm{ex}}\left[\rho \right]=-\frac{1}{2}\int a\left(\rho \right)\rho^{2}\;dr \end{array}$$

with

(A110) $$\begin{array}{c} a\left(\rho \right)=-\int u_{\mathrm{eff}}\left(r;\rho \right)\;dr. \end{array}$$

Therefore, the excess free energy density is given by

(A111) $$\begin{array}{c} f_{\mathrm{ex}}\left(\rho \right)=-\frac{1}{2}a\left(\rho \right)\rho^{2}. \end{array}$$

For short-range interactions, the excess free energy is usually proportional to the temperature *T* so that

(A112) $$\begin{array}{c} f_{\mathrm{ex}}\left(\rho \right)=\frac{k_{B}T}{m}\varphi_{\mathrm{ex}}\left(\rho \right), \end{array}$$

where $\varphi_{\mathrm{ex}}\left(\rho \right)$ is a function that depends only on the density. In that case, the total free energy $F=F_{\mathrm{id}}+F_{\mathrm{ex}}$ can be written as

(A113) $$\begin{array}{c} F\left[\rho \right]=\frac{k_{B}T}{m}\int C\left(\rho \right)\;dr, \end{array}$$

where

(A114) $$\begin{array}{c} C\left(\rho \right)=\rho \left[\ln\left(\lambda^{3}\frac{\rho }{m}\right)-1\right]+\varphi_{\mathrm{ex}}\left(\rho \right). \end{array}$$

Correspondingly, the pressure is given by

(A115) $$\begin{array}{c} P\left(\rho \right)=P_{\mathrm{id}}\left(\rho \right)+P_{\mathrm{ex}}\left(\rho \right)=\rho \frac{k_{B}T}{m}+\sigma_{\mathrm{ex}}\left(\rho \right)\frac{k_{B}T}{m}=\sigma \left(\rho \right)\frac{k_{B}T}{m}, \end{array}$$

where $\sigma_{\mathrm{ex}}\left(\rho \right)=\varphi_{\mathrm{ex}}^{\prime }\left(\rho \right)\rho -\varphi_{\mathrm{ex}}\left(\rho \right)$ and $\sigma \left(\rho \right)=\rho +\sigma_{\mathrm{ex}}\left(\rho \right)$ (see Appendix A). This is a manner to justify from a microscopic model the generalized free energy introduced in Section 2. If we now add a potential of long-range interactions (using a mean field approximation) we recover the model treated in this paper with g(ρ)=ρ.
